# Blood-Based Protein Biomarkers for the Management of Traumatic Brain Injuries in Adults Presenting to Emergency Departments with Mild Brain Injury: A Living Systematic Review and Meta-Analysis

**DOI:** 10.1089/neu.2017.5182

**Published:** 2021-04-05

**Authors:** Stefania Mondello, Abayomi Sorinola, Endre Czeiter, Zoltán Vámos, Krisztina Amrein, Anneliese Synnot, Emma Donoghue, János Sándor, Kevin K.W. Wang, Ramon Diaz-Arrastia, Ewout W. Steyerberg, David K. Menon, Andrew I.R. Maas, Andras Buki

**Affiliations:** ^1^ Department of Biomedical and Dental Sciences and Morphofunctional Imaging, University of Messina, Messina, Italy.; ^2^ Department of Neurosurgery, University of Pecs, Pecs, Hungary.; ^3^ János Szentágothai Research Centre, University of Pecs, Pecs, Hungary.; ^5^ Anesthesiology and Intensive Therapy, University of Pecs, Pecs, Hungary.; ^4^ MTA-PTE Clinical Neuroscience MR Research Group, Pecs, Hungary.; ^6^ Australian & New Zealand Intensive Care Research Centre (ANZIC-RC), Monash University, Melbourne, Victoria, Australia.; ^7^ Cochrane Consumers and Communication Group, Centre for Health Communication and Participation, La Trobe University, Melbourne, Victoria, Australia.; ^8^ Department of Preventive Medicine, Division of Biostatistics and Epidemiology, University of Debrecen, Debrecen, Hungary.; ^9^ Program for Neuroproteomics & Biomarkers Research, Departments of Psychiatry & Neuroscience, McKnight Brain Institute, University of Florida, Gainesville, Florida.; ^10^ Department of Neurology, University of Pennsylvania Perelman School of Medicine, Philadelphia, Pennsylvania.; ^11^ Center for Clinical Decision Sciences, Department of Public Health, Erasmus Medical Center, Rotterdam, The Netherlands.; ^12^ Division of Anaesthesia, University of Cambridge, Cambridge, United Kingdom.; ^13^ Department of Neurosurgery, Antwerp University Hospital and University of Antwerp, Antwerp, Belgium.

**Keywords:** biomarkers, diagnosis, living systematic review, meta-analysis, TBI

## Abstract

Accurate diagnosis of traumatic brain injury (TBI) is critical to effective management and intervention, but can be challenging in patients with mild TBI. A substantial number of studies have reported the use of circulating biomarkers as signatures for TBI, capable of improving diagnostic accuracy and clinical decision making beyond current practice standards.

We performed a systematic review and meta-analysis to comprehensively and critically evaluate the existing body of evidence for the use of blood protein biomarkers (S100 calcium binding protein B [S100B], glial fibrillary acidic protein [GFAP], neuron specific enolase [NSE], ubiquitin C-terminal hydrolase-L1 [UCH-L1]. tau, and neurofilament proteins) for diagnosis of intracranial lesions on CT following mild TBI. Effects of potential confounding factors and differential diagnostic performance of the included markers were explored. Further, appropriateness of study design, analysis, quality, and demonstration of clinical utility were assessed. Studies published up to October 2016 were identified through searches of MEDLINE^®^, Embase, EBM Reviews, the Cochrane Library, World Health Organization (WHO), International Clinical Trials Registry Platform (ICTRP), and clinicaltrials.gov. Following screening of the identified articles, 26 were selected as relevant. We found that measurement of S100B can help informed decision making in the emergency department, possibly reducing resource use; however, there is insufficient evidence that any of the other markers is ready for clinical application. Our work pointed out serious problems in the design, analysis, and reporting of many of the studies, and identified substantial heterogeneity and research gaps. These findings emphasize the importance of methodologically rigorous studies focused on a biomarker's intended use, and defining standardized, validated, and reproducible approaches. The living nature of this systematic review, which will summarize key updated information as it becomes available, can inform and guide future implementation of biomarkers in the clinical arena.

*Editor's Note: This article is published as a Living Systematic Review. All Living Systematic Reviews will be updated at approximately three-six month intervals, with these updates published as supplementary material in the online version of the* Journal of Neurotrauma (see Update).

## Introduction

Traumatic brain injury (TBI) is among the most common neurological disorders worldwide, and globally, its incidence continues to rise.^1,2^ According to the Centers for Disease Control (CDC) in the United States, over the past decade, rates of TBI-related emergency department (ED) visits have increased by 70%. Most of these TBIs are classified as mild (mTBI), posing a substantial everyday workload. Clinical diagnosis remains a challenge, and CT is considered the diagnostic cornerstone used in the ED to rule out post-traumatic brain lesions and complement clinical assessment of patients with a possible mTBI.^3^ However, it is generally acknowledged that CT is not always available, implies patient radiation exposure, and is relatively costly in terms of ED logistical burden and healthcare expenditures because of the small proportion of subjects (∼10%) diagnosed as having actual traumatic intracranial lesions.^3,4^

The need to manage patients with possible mTBI more effectively and efficiently–to reduce unnecessary CT scans and medical costs, while not compromising patient care and safety–has driven the quest for sensitive blood-based markers as objective parameters that can be easily and rapidly measured in the systemic circulation. Identification of biomarker signatures associated with distinct aspects of TBI pathophysiology may be also of clinical value for a more accurate characterization and risk stratification of TBI, thereby optimizing medical decision making and facilitating individualized and targeted therapeutic intervention. As such, over the past decades, a focused effort has been made to identify novel blood biomarkers for TBI, and a growing number of candidates has been described and proposed,^5–8^ leading to the recent incorporation of S100B into the Scandinavian Neurotrauma Guidelines.^9^ Nonetheless at present, the role of body fluid biomarkers in TBI is primarily relegated to research studies, and the provision of high quality evidence is paramount to meet regulatory requirements and support their adoption and routine use in clinical practice.

Meta-analysis can exploit the quantity of data collected in separate studies and provide the statistical power to assess more precise estimates of sensitivity and specificity, to determine influence of potential confounding factors on the biomarker diagnostic performance, and to detect differences in the accuracy of different marker tests. Hence, we conducted a systematic review and meta-analysis to comprehensively summarize and critically evaluate the existing body of evidence for the use of blood protein biomarkers for diagnosis of brain injury as assessed by CT in adult patients presenting to the ED after mild head trauma.

We focused on markers for which promising scientific evidence of analytical and clinical validity is available and which therefore, are likely to be rapidly transferable to clinical practice; namely, S100 calcium binding protein B (S100B), glial fibrillary acidic protein (GFAP), neuron specific enolase (NSE), ubiquitin C-terminal hydrolase-L1 (UCH-L1), and tau and neurofilament proteins. As TBI biomarker research and technological and analytical advances are dynamic, we felt that a living systematic review–a high quality, online review that is updated as new research becomes available^10^–would best fit our purpose. The “*living*” nature of such work will permit the potential inclusions and investigation of novel markers, marker combinations, and more refined diagnostic time windows for which relevant scientific literature/body of evidence will be gained.

## Methods

This review is being prepared as a “living systematic review,” initiated in the context of the CENTER-TBI project (www.center-tbi.eu).^10–12^ Following a predefined protocol registered on the PROSPERO database (registration number CRD42016048154), we conducted a systematic review and meta-analysis according to the Preferred Reporting Items for Systematic Reviews and Meta-Analyses (PRISMA) guidelines.^13^

### Information sources

We searched Ovid MEDLINE^®^ (1946 to October 2016), OVID Embase (1980 to October 2016), OVID Evidence-Based Medicine (EBM) Reviews (October 2016) and Cochrane Library (October 2016) for relevant studies. The search strategies used can be found in the supplementary Appendix (see online supplementary material at http://www.liebertpub.com).

For possible ongoing trials and studies, we searched the World Health Organization (WHO) International Clinical Trials Registry Platform (ICTRP) (searched November 2016) and ClinicalTrials.gov registry (searched November 2016). Update searches will be run every 3 months after publication, to identify new studies for inclusion in this living systematic review.

Additional studies were identified by reviewing the reference lists of published clinical trials and relevant narratives as well as systematic reviews. Abstracts from relevant scientific meetings were also examined, and experts in the field were consulted for any further studies.

Citations were uploaded into a web-based systematic review program (Covidence, Alfred Health Melbourne, Australia) (http://www.covidence.org/).

### Study selection

Two reviewers independently reviewed the title and abstract of each citation identified by the search strategy. In the second stage, the full text was reviewed and eligible studies selected. Any disagreement between the two authors was resolved through discussion, or where necessary, arbitration by a third party. Studies were included if the article met the prespecified list of eligibility criteria: studies enrolling adult patients presenting to the ED with a history of possible brain injury complying with any authors' definition of mTBI; report of the admission head CT findings; at least one quantitative measurement of the circulating biomarkers of interest (S100B, GFAP, NSE, UCH-L1, tau, and neurofilament proteins) on admission; and relevant accuracy data.

We included studies containing mixed populations; that is, participants with moderate and severe TBI (Glasgow Coma Score [GCS] <13) or pediatric populations. Studies were included irrespective of their geographic location and language of publication. We excluded studies using non-quantitative methods to assess biomarker concentrations (e.g., Western blot or explorative proteomics). Studies with small cohorts (< 50 participants) were excluded, given the high likelihood of their being underpowered and therefore impacting the reliability of findings.

### Data extraction and assessment of methodological quality

Two reviewers independently extracted data using a standardized data abstraction form. We abstracted relevant information related to the study design, patient characteristics (demographic and clinical data, including indices of injury severity, presence of extracerebral injuries and polytrauma, and CT findings) and biomarker characteristics (concentrations, sampling time, cutoffs, and statistical levels of diagnostic accuracy [sensitivity and specificity]), analytical aspects of biomarker testing, and study limitations. Details regarding the definition of mTBI and CT abnormality were also extracted.

In the case of multiple studies from the same research group, authors were contacted to ensure that there was no overlap in patient populations. We also contacted authors for clarification of study sample, missing data, or ambiguity in the cutoffs used. If biomarker measurements were taken at multiple time points, we used the sample on admission for analysis.

The methodological quality of the included studies was independently assessed by two reviewers using a modified version of the tool for quality assessment of studies of diagnostic accuracy included in systematic reviews (QUADAS-2),^14^ as recommended by the Cochrane Collaboration. Discrepancies were resolved through discussion or arbitration by a third reviewer.

### Statistical analysis and data synthesis

The analysis includes a structured narrative synthesis. We constructed evidentiary tables identifying the results pertinent to diagnostic capabilities of the different biomarkers (detection of intracranial lesions as assessed by CT) and study characteristics for all included studies. We conducted exploratory analyses by plotting estimates of sensitivity and specificity from each study on forest plots and in receiver operating characteristic (ROC) space.

Where adequate data were available, we performed meta-analyses for each biomarker, to summarize data and obtain more precise estimates of diagnostic performance. For studies with diverse thresholds, we meta-analyzed pairs of sensitivity and specificity using the hierarchical summary ROC (HSROC) model, which allows for the possibility of variation in threshold between studies, and also accounts for variation among studies and any potential correlation between sensitivity and specificity.^15^ For these analyses, we used the NLMIXED procedure in SAS software (version 9.4; SAS Institute 2011, Cary, NC). For studies that reported data at common prespecified cutoff values, we calculated the pooled estimates of sensitivity and specificity (clinically interpretable), by undertaking a random effects bivariate regression approach.^16^

We explored heterogeneity through visual examination of the forest plot and the SROC plot for each biomarker. However, as there were insufficient studies, lack of individual data, and/or important variation across studies with simultaneous presence of factors with potentially diverging effects on biomarker accuracy estimates, we did not perform meta-regression (by including each potential source of heterogeneity as a covariate in the bivariate model) as planned.

Sensitivity analyses were performed to check the robustness of the results. We used Cook's distance to identify particularly influential studies, and checked for outliers using scatter plots of the standardized predicted random effects. Then, the robustness of the results was checked by refitting the model excluding any outliers and very influential studies. Sensitivity analyses were also conducted to investigate the impact on biomarker performance of studies including mixed populations, bias in the selection of participants, high prevalence of abnormal CT findings, and different definitions of TBI as assessed by CT.

Data processing and statistical analyses were conducted using Review Manager version 5.3 (Cochrane Collaboration, Copenhagen, Denmark) and STATA version 13.0 (StataCorp, Colleage Station, TX) including the user written commands METANDI and MIDAS.

### Quality of the evidence

The Grading of Recommendations Assessment, Development, and Evaluation (GRADE)^17^ approach was used to assess the overall quality of evidence of the included biomarker tests. The results were summarized using GRADEPro software (Version 3.2, 2008).

## Results

### Description of studies

**FIG. 1.**
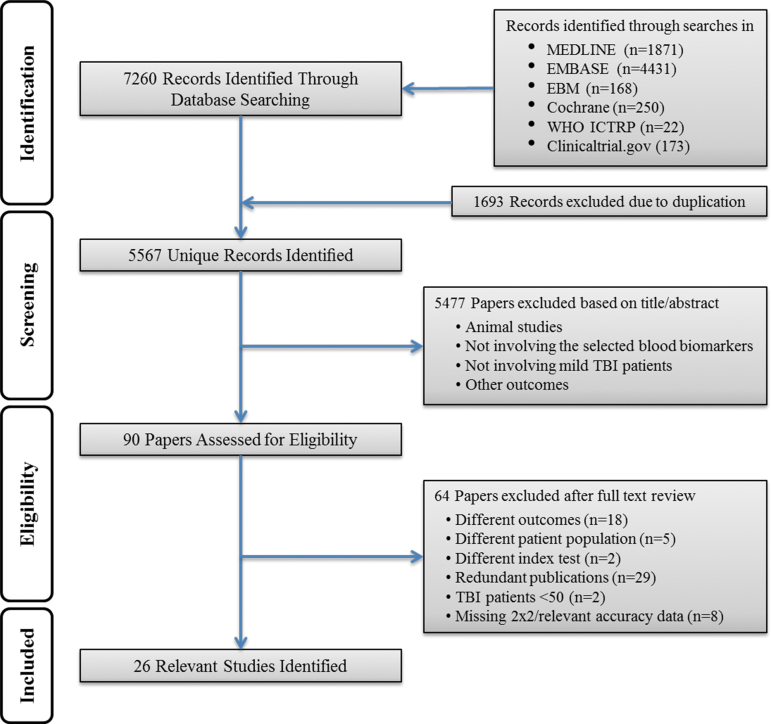
Study flow diagram.

Our search strategy identified a total of 7260 citations. Removal of duplicates resulted in 5567 distinct citations, of which 90 full-text articles were assessed for eligibility, and 26 articles^3,18–42^ were included in the systematic review (Fig. 1, flow diagram of search and eligibility results, and Table 1). Tables 2 and 3 show the main characteristics of the included publications, and additional details are provided in Tables S1 and S2(see online supplementary material at http://www.liebertpub.com).

**Table 1.** Summary of the Number and Characteristics of Primary Articles Identified for Each Biomarker

Marker	No. of studies	No. of participants	No. of studies (%) by no. of participants in each study		No. of studies by GCS		No. of studies with predefined cutoff	No of studies by sample type	Relevant results (Range individual sensitivities and specificities)
S100B	22	7754 (CT+ = 713; CT- = 7041)	50–100	4 (18)	GCS 15:	1	16	Serum 21	Sens 0.83–1.00
			101–200	7 (32)	GCS 14–15:	3		Plasma 1	Spec 0.12–0.77
			201–500	6 (27)	GCS 13–15:	15			
			>500	5 (23)	GCS 9–15:	2			
					GCS 3–15:	1			
GFAP	4	783 (CT+ = 198; CT- = 595)	101–200	1 (25)	GCS 9–15:	3	0	Serum 3	Sens 0.67–1.00
			201–500	3 (75)	GCS 3–15:	1		Plasma 1	Spec 0.00–0.89
NSE	3	314 (CT+ = 55; CT- = 259)	50–100	1 (33)	GCS 14–15:	1	0	Serum 3	Sens 0.56–1.00
			101–200	2 (67)	GCS 13–15:	2			Spec 0.07–0.77
UCH-L1	2	347 (CT+ = 64; CT- = 283)	50–100	1 (50)	GCS 9–15:	2	0	Serum 2	Sens 1.00
			201–500	1 (50)					Spec 0.21–0.39
Tau	1	50 (CT+ = 10; CT- = 40)	50–100	1 (100)	GCS 13–15:	1	0	Serum 1	Sens 0.50
									Spec 0.75

GCS, Glasgow Coma Scale; S100B, S100 calcium binding protein B; GFAP, glial fibrillary acidic protein; NSE, neuron specific enolase; UCH-L1, ubiquitin C-terminal hydrolase-L1.

**Table 2.** Characteristics of the 26 Included Studies

Study ID	BM	No TBI	GCS	Inclusion criteria	Prevalence of positive CT scan findings	Age (years)^*^	Sex (% female)	Polytrauma/ ECI
Asadollahi 2016^18^	S100B	158	13–15	History of isolated mTBI, age ≥18 yr., admission within 2 h of injury	50%	35.4 (15.8)	48 (30.4%)	No
Bazarian 2013^19^	S100B	787	13–15	GCS >13 measured 30’ or more after injury, patient age ≥1 yr., blood drawn within 6 h of injury, CT scan performed as part of the clinical care	6%	38.2 (19.5) Children & adolescents included	287 (36.5%)	Yes
Biberthaler 2001^20^	S100B	52	13–15	History of isolated MHT, GCS 13–15, at least one of the following symptoms: amnesia, LOC, nausea, vomiting, vertigo, or severe headache	29%	NR	14 (27%)	No
Biberthaler 2006^3^	S100B	1309	13–15	History of isolated head trauma, admission within 3 h, GCS 13–15 on admission, at least one of the following risk factors: LOC, PTA, nausea, vomiting, severe headache, dizziness, vertigo, intoxication, anticoagulation, age >60 yr.	7%	Median (IQR) 47 (32–75)	454 (35%)	No
Bouvier 2009^21^	S100B	105	13–15	History of isolated head trauma and admission within 3 h, GCS 13–15 on admission, at least one of the following risk factors: LOC, PTA, nausea, vomiting, severe headache, dizziness, vertigo, intoxication, anticoagulation, age >60 yr.	15%	53 (range 18–94; IQR 37)	40 (38%)	No
Calcagnile 2012^22^	S100B	512	14–15	History of head trauma, GCS 14–15 during examination and LOC <5’ and/or amnesia	5%	42.2	198 (38.5%)	Unclear
Cervellin 2012^23^	S100B	60	14–15	History of MHI, GCS 14–15 on admission, patients with chronic neurological diseases, but not those with suspected/visible brain tumor	33%	58 (range 14–80) Adolescents included	18 (32%)	No
Cervellin 2014^24^	S100B NSE	68	14–15	History of MHI, GCS 13–15 at admission, age >14 yr.	16%	55 (range 15–86) Adolescents included	24 (35%)	Unclear
Egea-Guerrero 2012^25^	S100B	143	15	Patient age ≥14 yr., GCS 15 at hospital admission and one or more of the following symptoms: transitory LOC; amnesia; persistent headache; nausea or vomiting; and vertigo	10.5%	49 (20.6) Including pediatric population >14	54 (37.8%)	Yes
Ingebrigtsen 2000^26^	S100B	182	13–15	Brain injury with brief LOC, GCS 13–15 at admission, age 15–80 yr., admission within 12 h post-injury, CT performed within 24 h after injury	5%	33 (range 15–78) Adolescents included	71 (39%)	Unclear
Laribi 2014^27^	S100B	431	13–15	History of isolated MHI; GCS 13–15 with one or more of the following: amnesia, LOC, nausea, vomiting, vertigo, anticoagulation before injury or severe headache on admission. Patient age ≥18 yr, admission within 3 h after injury	6%	Median (IQR) 36 (24–54)	152 (35%)	No
Ma 2008^28^	Tau	50	13–15	Patient age ≥18 yr., GCS 13–15 at admission, admission within 12 h of injury, CT performed as part of the clinical care, blunt head trauma followed by LOC and/or PTA	20%	40.3 (17.7)	12 (24%)	Unclear
McMahon 2015^29^	GFAP	215	3–15	Admission within 24 h of injury, positive clinical screen for acute TBI necessitating a noncontrast head CT according to ACEP/CDC evidence-based joint practice guidelines	51%	42.1 (18) (range 16–93)	54 (27%)	Yes
Morochovic 2009^30^	S100B	102	13–15	Patients with brain injury, GCS 13–15 with or without risk factors	18%	42.0 (19.7) (range 12–84) Including pediatric population	31 (30.39%)	Yes
Muller 2007^31^	S100B	236	13–15	History of brain injury; LOC or PTA; GCS 13–15 at admission; CT scan within 12 h of trauma	9%	39 (range 18–92)	58 (25.7%)	No
Muller 2011^32^	S100B	233	13–15	Adult patients (>16yr.), GCS 13–15	9%	Median (IQR) 48.4 (24–72) (range 11–97) Adolescents included	90 (39%)	No
Mussack 2002^33^	S100B NSE	139	13–15	History of trauma, GCS 13–15, and at least one of the following symptoms: transient LOC (less than 5’), PTA, nausea, vomiting, or vertigo	14%	Median 36.0	33 (24%)	No
Papa 2012^34^	GFAP	307	9–15	History of blunt head trauma followed by LOC, amnesia, or disorientation; GCS 9–15; admission to the ED within 4 h of injury; patient age ≥18 yr.	30%	39 (15) (range 18–89)	38 (35%)	Unclear
Papa 2012^35^	UCH-L1	96	9–15	History of blunt head trauma followed by LOC, amnesia, or disorientation; GCS 9–15; admission to the ED within 4 h of injury; patient age ≥18 yr.	29%	39 (15) (range 18–89)	36 (38%)	Unclear
Papa 2014^36^	S100B GFAP		9–15	History of blunt head trauma followed by LOC, amnesia, or disorientation; GCS 9–15; admission to the ED within 4 h of injury; patient age ≥18 yr.	10%	40 (16)	78 (37%)	Yes
Poli-de-Figueiredo 2006^37^	S100B	50	13–15	Isolated MHI, GCS 13–15, at least one of the following symptoms: amnesia, LOC, nausea, vomiting, vertigo, or severe headache	12%	NR	22 (44%)	No
Romner 2000^38^	S100B	278	3–15	Brain injury with LOC, blood sample collected within 24 h after injury, and CT performed within 24 h after the injury. LOC was considered to have occurred when the patient had amnesia for the trauma event and if accompanying persons reported LOC.	9%	32 (range 1–84) Children & adolescents included	103 (37%)	Yes
Thaler 2015^39^	S100B	782	13–15	MHI (GCS Score 13–15) in patients on medication with PAI with age ≥18 yr., and MHI in patients with age ≥65 yr. independent of PAI intake; admission within 3 h of injury	6%	Median 83 (range 74–88)	537 (68.7%)	No
Welch 2016^40^	S100B GFAP UCH-L1	251	9–15	GCS 9–15 on admission, patient age ≥18 <80 yr.; acceleration or deceleration closed injury to the head; admission within 4 h after injury; ED workup included a head CT scan.	14%	45.6 (18.4) (range 18–80)	100 (39.8%)	Unclear
Wolf 2013^41^	S100B NSE	107	13–15	GCS 13–15 at admission, blunt head trauma, admission to the ED within 3 h of injury	23%	59 (23) (range 18–97)	47 (44%)	No
Zongo 2012^42^	S100B	1560	13–15	Patient age ≥15 yr., GCS 13–15, admission to the ED within 6 h of injury, at least one of the following risk factors: LOC, PTA, repeated vomiting, severe headache, dizziness, vertigo, alcohol intoxication, anticoagulation, and age >65 yrs.	7%	median (IQR) 57 (32–82) Adolescents included	690 (44.2%)	No

^*^ Mean (SD) unless stated otherwise.

ACEP/CDC, American College of Emergency Physicians/ Centers for Disease Control and Prevention; BM, biomarker; ECI, extracranial injury; ED, emergency department; GCS, Glasgow Coma Scale; GFAP, glial fibrillary acidic protein; IQR, interquartile range; LOC, loss of consciousness; MHI, mild head injury; MHT, mild head trauma; mTBI, mild traumatic brain injury; NR, not reported; NSE, neuron specific enolase; PAI, platelet aggregation inhibitor; PTA, post-traumatic amnesia; S100B, S100 calcium binding protein B; UCH-L1, ubiquitin C-terminal hydrolase-L1.

**Table 3.** Biomarker Characteristics of the 26 Included Studies

Study ID	Sampling type	Assay analyzer & manufacturer/is	Timing of sample collection^a^	Assay range/ CV	Cutoff	BM levels in TBI patients^c^	BM levels in patients with CT positive^c^	BM levels in patients with CT negative^c^	BM levels in controls^c^
*S-100B*									
Asadollahi 2016^18^	Serum (venous)	ECLIA Elecsys^®^ Roche	Within 3 h post-injury	LOD 0.02μgL range 0.02–30 CV <10%	0.11 μg/L	NR	Mean (95% CI) 0.68 μgL (0.58–0.77)	Mean (95% CI) 0.10 μgL (0.07–0.11)	NA
Bazarian 2013^19^	Serum (venous)	ECLIA Elecsys^®^ Roche	Within 6 h post-injury	Range 0.005-39 μgL	0.10 μg/L^b^	0.149 μg L	0.292 μg/L	0.144 μg/L	0.071 μg/L Negative control group
Biberthaler 2001^20^	Serum (venous)	ILMA LIA-mat, Sangtec 100	On admission 116’ (18.8)	NR	0.10 μg/L	Mean (SD) 0.470 ng/mL (0.099)	NR	NR	0.05 ng/mL (0.01) Negative control group 7.16 ng/mL (3.77) Positive control group
Biberthaler 2006^3^	Serum (venous)	ECLIA Elecsys^®^ Roche	Within 3 h post-injury Median 60’ (range 40–80')	LOD 0.005 μgL range 0.005–39	0.10 μg/L	0.17 μg/L (0.10–0.37)	0.49 μg/L (0.25–1.46)	0.16 μg/L (0.09–0.33)	0.05 μg/L (0.03–0.06) Negative control group 0.45 μg/L (0.19–2.63) Positive control group
Bouvier 2009^21^	Serum (venous)	ECLIA Elecsys^®^ Roche	On admission Median 1 h 36’	LOD 0.005 μgL range 0.005–39	0.10 μg/L^b^	Mean 0.37 μg/L (SD 0.76)	Mean 0.88 μg/L (SD 1.52)	Mean 0.28 μg/L (SD 0.49)	Mean (SD) 0.05 μg/L (0.02) Negative control group
Calcagnile 2012^22^	Serum (venous)	ECLIA Elecsys^®^ Roche	Within 3 h post-injury	LOD 0.005 μgL range 0.005–39 Intra-assay CV <2.1%	0.10 μg/L	NR	NR	NR	NA
Cervellin 2012^23^	Serum (venous)	ILMA Liaison^®^ Diasorin	Within 3 h post-injury 62’	LOD 0.02- μg/L range 0.02–30 CV <10%	0.38 μg/L	NR	Geometric mean 1.35 μg/L (95% CI 0.73–1.97)	Geometric mean 0.48 μg/L (95% CI 0.33–0.63)	NA
Cervellin 2014^24^	Serum (venous)	ILMA Liaison^®^ Diasorin	Within 3 h post-injury 62’	LOD 0.02- μg/L range 0.02–30 CV <10%	0.56 μg/L	NR	1.5 μg/L (1.19–2.37)	0.22 μg/L (0.12–0.48)	NA
Egea-Guerrero 2012^25^	Serum (venous)	ECLIA Elecsys^®^ Roche	Within 6 h post-injury	LOD 0.005μgL range 0.005–39	0.105μg/L^b^	Mean (95% CI) 0.392 μg/L (0.327–0.456)	Mean (95% CI) 0.585 μg/L (0.363–0.806) Median 0.350	Mean (95% CI) 0.369 μg/L (0.302–0.436) Median 0.220	NA
Ingebrigtsen 2000^26^	Serum (venous)	RIA AB Sangtec	On admission 3 h (range 0.5–12.0)	LOD 0.2 μg/L	0.2 μg/L	Mean 0.5 μg/L (range 0.2–1.9) Detectable in 69 (38%) pts, undetectable in 113 (62%)	Mean 0.7 μg/L (range 0.2–1.9) 9/10 with detectable level	NR	NA
Laribi 2014^27^	Serum (venous)	ECLIA Elecsys^®^ Roche	Within 3 h post-injury Median (IQR) 115’ (75–150)	LOD 0.005 μgL range 0.005–39 Intra-assay CV 2.1% Inter-assay CV 2.8%	0.10 μg/L	H0 - 0.14 μg/L (0.08–0.25) H+3 - TBI 0.10 μg/L (0.06–0.16)	H0 - 0.24 μg/L (0.15–0.34) H+3 - 0.13 μg/L (0.10–0.25)	H0 - 0.13 μg/L (0.08–0.25) H+3 - 0.10 μg/L (0.06–0.15)	NA
Morochovic 2009^30^	Serum (venous)	ECLIA Elecsys^®^ Roche	Within 3 h post-injury 1.8 h	LLOD 0.005 μg/L Inter-assay CV 4.9%	0.10 μg/L	Mean (SD) GCS 13 0.26 μg/L (0.34) GCS 14 0.43 μg/L (0.56) GCS 15 0.85 μg/L (3.11)	NR	NR	NA
Muller 2007^31^	Serum (venous)	ILMA Liaison^®^ Diasorin	Within 12 h post-injury	LOD 0.013 μg/L Intra-assay CV <5% Inter-assay CV <10%	0.10 μg/L	Mean (95% CI) GCS 13 0.32 μg/L (0.16–0.49) GCS 14 0.22 μg/L (0.13–0.30) GCS 15 0.18μg/L (0.16–0.21)	Mean (95% CI) 0.36 μg/L (0.21–0.50)	Mean (95% CI) 0.18 μg/L 0.16–0.20	NA
Muller 2011^32^	Serum (venous)	ECLIA Elecsys^®^ Roche	NR	NR	0.105 μg/L^b^	NR	NR	NR	NA
Mussack 2002^33^	Serum (venous)	ILMA Liaison^®^ Diasorin	On admission Median 24.3’	LLOD 0.02 ng/mL	0.21 ng/mL	0.24 ng/mL (0.15–0.49)	0.94 ng/mL (0.39–1.43)	0.22 ng/mL (0.14–0.39)	0.06 ng/mL (0.05–0.09) Negative control group
Papa 2014^36^	Serum (venous)	ELISA Banyan	Within 4 h post-injury 3.1 h (95% CI 2.8–3.3)	LLOQ 0.083 ng/mL LLOD 0.017 ng/mL	0.020 ng/mL	NR	NR	NR	NR
Poli-de-Figueiredo 2006^37^	Serum (venous)	ECLIA Elecsys^®^ Roche	On admission Median (IQR) 82’ (60–110)	NR	0.10 μg/L	0.29 μg/L (0.14–0.76)	0.75 μg/L (0.66–6.5)	0.26 μg/L (0.12–0.65)	0.04 μg/L (0.03–0.05) Negative control group
Romner 2000^38^	Serum (venous)	RIA Sangtec	Within 24h post-injury 3.8 h (range 0.5–24.0)	LOD 0.2 μg/L	0.2 μg/L (LOD)	Mean 0.6 m g/L (range 0.2–6.2) Detectable in 35% MHI	Mean 2.2 μg/L (range 0.2–12.5) Detectable in 23 (92%) mild-severe TBI pts	NR	Non detectable levels Negative control group
Thaler 2015^39^	Serum (venous)	ECLIA Elecsys^®^ Roche	Within 3 h post-injury Median (IQR) 2.05h (1.30–2.30)	Range 0.005–39 μgL	0.105 μg/L	mTBI 0.15μg/L (0.088–0.291) GCS 15 0.139 (0.085–0.267) GCS 14 0.178 (0.102–0.311) GCS 13 0.284 (0.130–0.652)	0.285 μg/L (0.185–0.532)	0.143 μg/L (0.085–0.274)	NA
Welch 2016^40^	Serum (venous)	ECLIA Cobas 6000^®^ Roche	Within 6 h post-injury	NR	0.10 μg/L^b^	120 (70–230) All values in detectable range	NR	NR	NA
Wolf 2013^41^	Serum (venous)	ECLIA Elecsys^®^ Roche	Within 3 h post-injury	NR	0.105 μg/L^b^	NR	Mean (SD) 0.7 μg/L (1.19)	Mean (SD) 0.21 μg/L (0.26)	NA
Zongo 2012^42^	Plasma (venous)	ECLIA Elecsys^®^ Roche	Within 6 h post-injury	NR	0.10 μg/L^b^	0.23 μg/L (0.14–0.38)	0.46 μg/L (0.27–0.72)	0.22 μg/L (0.14–0.36)	NA
*GFAP*									
McMahon 2015^29^	Plasma (venous)	ELISA Banyan	Within 24 h post-injury	LLOD 0.01 ng/mL Intra-assay CV 4.3–7.8% Inter-assay CV 7.8–14.3%	0.6 ng/mL	NR	Mean (SD) 2.86 ng/mL (3.74)	Mean (SD) 0.26 ng/mL (0.41)	NA
Papa 2012^34^	Serum (venous)	ELISA Banyan	Within 4 h post-injury 2.6 h (95% CI 2.4–2.9)	LLOD 0.020 ng/mL Intra-assay CV 4.3–7.8%, Inter-assay CV 7.8–14.3%	0.035 ng/mL	0.316 ng/mL (IQR 0.60) Mean (SD) 0.893 (1.677) (95% CI 0.573 – 1.213)	NR	NR	0.010 ng/mL (0.050) Negative control group 0.216 ng/mL (0.275) Orthopedic control group 0.122 ng/mL (0.373) MVA control group 0.010 ng/mL (0.060) All controls
Papa 2014^36^	Serum (venous)	ELISA Banyan	Within 4 h post-injury 3.1 h (95% CI 2.8–3.3)	LLOQ 0.030 ng/mL ULOQ 50.000 ng/mL LLOD 0.008 ng/mL	0.067 ng/mL	NR	NR	NR	NR
Welch 2016^40^	Serum (venous)	ELISA Banyan	Within 6 h post-injury	NR	0 pg/mL	10.3 pg/mL (3.5, 37.4) 45 pts below LOD (4 with CT+)	NR	NR	NA
*NSE*									
Cervellin 2014^24^	Serum (venous)	IFMA Kryptor (BRAHMS AG)	Within 3 h post-injury 62’	LOD 0.08 μgL CV <6%	9.0 μg/L	NR	13.3 μg/L (12.1–20.3)	9.6 μg/L (8.2–12.3)	NA
Mussack 2002^33^	Serum (venous)	ECLIA Elecsys^®^ Roche	On admission Median 24.3’	LLOD 0.01 ng/mL	12.28 ng/mL	17.50 ng/mL (14.40–21.34)	18.43 ng/mL (15.31–26.03)	17.46 ng/mL (14.31–20.77)	15.55 ng/mL (14.90–17.00) Negative control group
Wolf 2013^41^	Serum (venous)	ECLIA Elecsys^®^ Roche	Within 3 h post-injury	NR	14.7 μg/L^b^	Missing values in 47 pts (44%)	Mean (SD) 18.1 μg/L (10.84)	Mean (SD) 12.4 μg/L (4.82)	NA
*UCH-L1*									
Papa 2012^35^	Serum (venous)	ELISA Banyan	Within 4 h post-injury 2.7 h (95% CI 2.4–2.9)	LLOD 0.030 ng/mL	0.029 ng/mL	Mean (SEM) 0.955ng/mL (0.248) (range 0.015–19.25)	Mean (SEM) 1.618 ng/mL (0.474)	Mean (SEM) 0.620 ng/mL (0.254)	Mean (SEM) 0.083 ng/mL (0.005) (range 0.015–0.490) All controls (Negative, orthopedic, MVA controls)
Welch 2016^40^	Serum (venous)	ELISA Banyan	Within 6 h post-injury	NR	40 pg/mL	65.8 (39.6, 125.2) 2 pts below LOD (none with CT+)	NR	NR	NA
*Tau*									
Ma 2008^28^	Serum (venous)	ELISA	On admission 5.0 h (2.8)	LOD 1.5 ng/mL	NR	Mean (SD) 5.0 ng/mL (2.98) 15 pts with detectable levels	NR	NR	NA

^a^ Mean (SD) unless stated otherwise.

^b^ Additional thresholds have been evaluated.

^c^ Median (IQR) unless stated otherwise.

Control group definition:

• Negative Control Group: healthy individuals (e.g., healthy volunteers, voluntary blood donors, outpatients for routine blood work) who were checked on their health and potential head trauma status.

• Positive Control Group: patients with moderate to severe brain injury.

• Orthopedic Control Group: non–brain-injured patients presenting to the ED with a single-limb orthopedic injury without blunt head trauma.

• MVA Control Group: patients presenting to the ED after a motor vehicle crash without blunt head trauma

BM, biomarker; CV, coefficient of variation; ECLIA, electrochemiluminescence immunoassay; ED, emergency department; ELISA, enzyme-linked immunosorbent assay; GCS, Glasgow Coma Score; GFAP, glial fibrillary acidic protein; H0, within 3 h after the clinical event; H+3, 3 h after the first sampling; IFMA, immunofluorometric assay; ILMA, immunoluminometric assay; IQR, interquartile range; LIA, luminescence immunoassay; LLOD, lower limit of detection; LLOQ, lower limit of quantification; LOD, limit of detection; mTBI, mild traumatic brain injury; MVA, motor vehicle accident; NA, not applicable; NR, not reported; NSE, neuron specific enolase; pts, patients; RIA, radioimmunoassay; S100B, S100 calcium binding protein B; SEM, standard error of the mean; TBI, traumatic brain injury; UCH-L1, ubiquitin C-terminal hydrolase-L1; ULOQ, upper limit of quantification.

Two of the 26 included articles reported biomarker results from the same patient cohort.^34,43^ All studies were published in 2000 or later. With the exception of one study published in French,^21^ and one published in Italian,^24^ all studies were published in English.

The total number of patients with TBI in the included studies was 8127, ranging from 50^28,37^ to 1560^42^ per study (median 170, interquartile range 104–258). Of those, 865 had positive CT scans, with an average prevalence of 17% (median 13%) (range 5–51%) (Table 2). Table S2 shows the criteria used for the definition of TBI/mTBI and positive CT scans (reference standard) in the different studies. In nine articles, the presence of a skull fracture was considered as a traumatic CT abnormality.

The reported mean or median age of the included patients ranged from 32^38^ to 83 years,^39^ with 10 studies including children and/or adolescents (patient age <18years). The total subject pool was largely male (median 63% across the studies), with the exception of the study by Thaler and colleagues, which was 68.7% female.^39^ Two cohort studies included mild to severe TBI patients (GCS 3–15),^29,38^ and two other cohorts included mild to moderate TBI patients (GCS 9–15).^34–36,40^ Six studies enrolled TBI patients with multiple trauma and/or extracranial injuries (Table 2). Nine of the included articles reported biomarker concentration from different types of control cohorts, including healthy individuals, or non–brain-injured trauma patients (See Table 3 for details).

Most of the studies defined the specific time frame from injury to blood draw as an inclusion criterion, with the majority of the samples collected within 6 h of injury (16 studies) and with mean or median time ranging from 24.3 min^33^ to 5 h (Table 3).^28^ In one study, samples were collected within 12 h,^31^ and in two studies, they were collected within 24 h.^29,38^

A single marker was evaluated in most of the studies (*n* = 21), while one study simultaneously assessed three markers.^40^ Of the eligible studies, 22 reported data on S100B (total number of TBI patients 7754), 4 reported data on GFAP (total number of TBI patients 783), 3 reported data on NSE (total number of TBI patients 314), and 2 reported data on UCH-L1 (total number of TBI patients 347). Fewer data were available for tau (one study that included only 50 patients),^28^ and we found no studies evaluating neurofilament proteins that met our inclusion criteria.

### Methodological quality

**FIG. 2.**
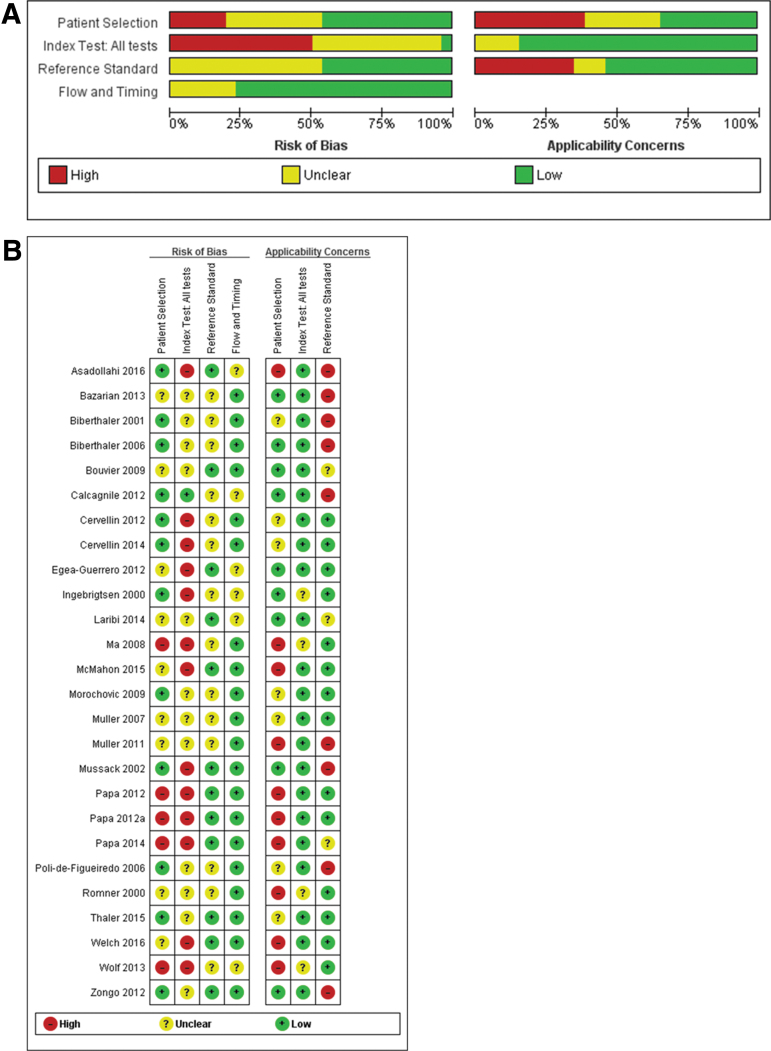
**(A)** Risk of bias and applicability concerns graph. Review authors' judgments about each domain presented as percentages across included studies. **(B)** Risk of bias and applicability concerns summary. Review authors' judgments about each domain for each included study.

The assessments of the methodological quality and risk of bias of the included studies are presented in Figure 2 and Figure S1(see online supplementary material at http://www.liebertpub.com). Participants neither consecutively nor randomly enrolled, the use of vague definitions of mTBI, or inclusion of an unrepresentative spectrum of patients (pediatric population or patients with GCS <13) may lead to incorporation bias, thus limiting the conclusions that can be drawn by affecting the accuracy estimates and compromising the applicability of the results.

In half of the studies, thresholds were not prespecified, and ROC analyses were used to determine optimal cutoffs, likely resulting in an overestimation of the diagnostic accuracy of the biomarker evaluated. In addition, the inclusion of skull fracture as a CT abnormality may cause inflation of the accuracy estimates of S100B, whereas, using a brain-specific marker as an index test may result in patients with skull fractures being misclassified as false negative. Finally, in different domains, a substantial number of studies were considered to be at unclear risk of bias because of substandard reporting. We investigated the effect of these factors in sensitivity and subgroup analyses.

### S100B

**FIG. 3.**
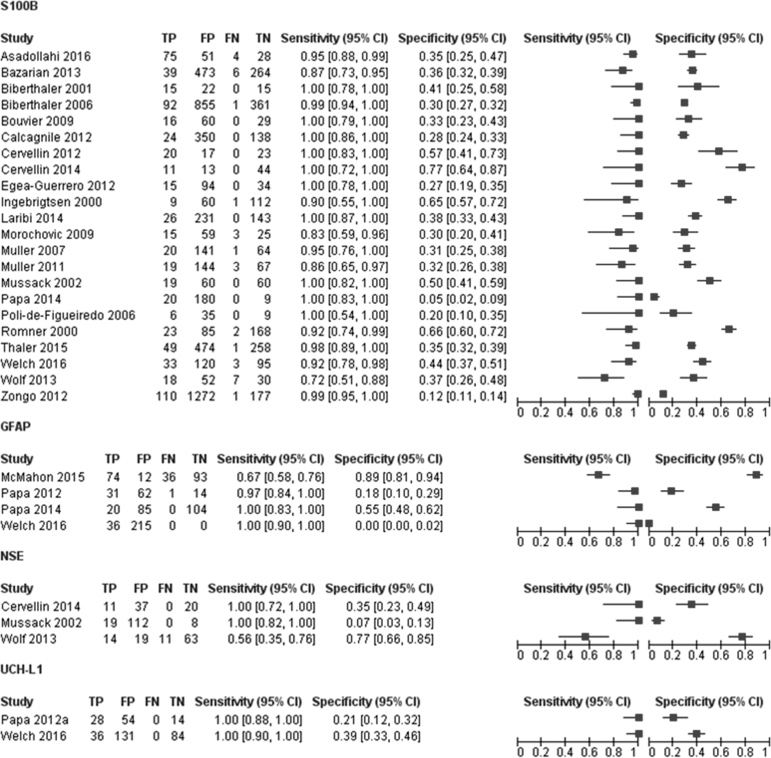
Forest plot showing individual sensitivity and specificity of circulating S100 calcium binding protein B (S100B), glial fibrillary acidic protein (GFAP), neuron specific enolase (NSE), and ubiquitin C-terminal hydrolase-L1 (UCH-L1) for detection of intracranial lesions on CT. Horizontal lines represent 95% confidence intervals. TP, true positive; FP, false positive; FN, false negative; TN, true negative.

**FIG. 4.**
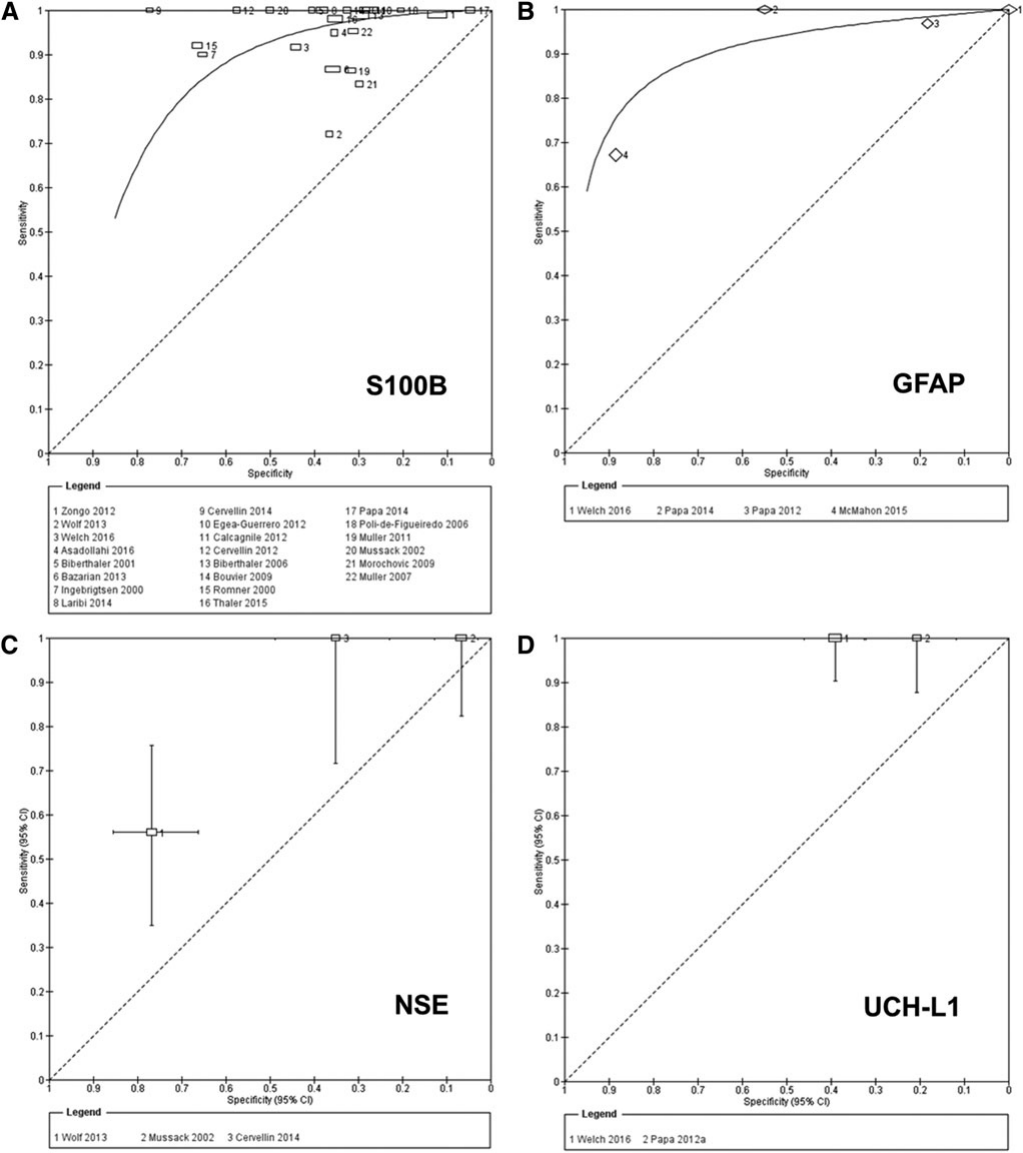
**(A, B)** Summary receiver operating characteristic (ROC) plots for S100 calcium binding protein B (S100B) and glial fibrillary acidic protein (GFAP) for detection of CT abnormalities. **(C, D)** Study estimates of sensitivity and specificity with 95% confidence intervals plotted in ROC space for neuron specific enolase (NSE), and ubiquitin C-terminal hydrolase-L1 (UCH-L1) for detection of CT abnormalities. Each square represents an individual study; the size of the symbol is proportional to the number of patients in each study. The hierarchical summary ROC (HSROC) model was used to estimate a summary curve using Proc NLMIXED in SAS.

The accuracy of S100B for detecting intracranial lesions on CT scan was evaluated in 22 studies (7754 patients).^3,18–27,30–33,36–42^ The individual sensitivities and the specificities were between 72% and 100% and between 5% and 77%, respectively (Fig. 3). All but six of the included studies used the same cutoff (0.10–0.11μg/L), which represents the 95th percentile of a healthy reference population and is conventionally considered to distinguish physiological from pathophysiological serum concentrations.^3^ Seven studies reported multiple cutoffs (Table 3). The summary ROC curve showing the accuracy of S100B across all the studies, regardless the threshold used, is presented in Figure 4.

In terms of the assays/platforms used, most of the studies (13/22) used an automated electrochemiluminescence immunoassay (ECLIA) on an Elecsys^®^ analyzer (Roche Diagnostics), while one used the Cobas 6000 analyzer (Roche Diagnostics). There were four studies conducted using an automated immunoluminometric assay (ILMA) on a Liaison^®^ analyzer (Diasorin), and one was conducted on LIA^®^-mat (Sangtec^®^ 100); one study used a radioimmunoassay (Sangtec), and one used an enzyme-linked immunosorbent assay (ELISA) platform (Banyan Biomarkers, Inc.) (Table 3). In one study, the analytical performance of the two automated immunoassays (i.e., Diasorin and Roche Diagnostics assays) was compared and, although not interchangeable, the two methods strongly correlated and appeared usable in a similar manner.^27^

### Performance of S100B at a 0.10–0.11μg/L cutoff value

**FIG. 5.**
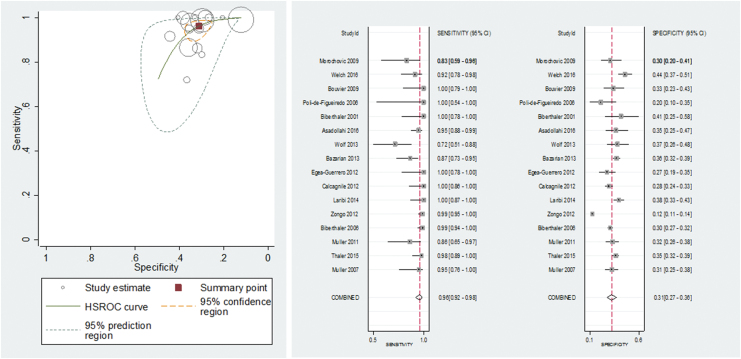
Summary receiver operating characteristics plot of sensitivity and specificity of S100 calcium binding protein B (S100B) at a 0.10–0.11μg/L cutoff value for detecting intracranial lesions on CT. Each circle represents an individual study; size of the symbol reflects the number of patients in the studies; red solid spot in the middle is summary sensitivity and specificity; inner ellipse represents 95% confidence region, and outer ellipse represents 95% prediction region.

To obtain clinically relevant estimates of the performance of S100B, we pooled the results from the 16 studies using the cutoff value of 0.10–0.11μg/L. The individual sensitivities and the specificities for each study included in this meta-analysis were between 72% and 100% and between 5% and 77%, respectively (Fig. 5). The following summary estimates were obtained: sensitivity 96% (95% CI 92–98%), specificity 31% (95% CI 27–36%), positive likelihood ratio 1.4 (1.3–1.5) and negative likelihood ratio 0.12 (0.06–0.25). Figure 5 shows the pooled sensitivity and specificity (the solid red spot in the middle) and the 95% confidence and prediction regions (the inner and outer ellipses, respectively).

There was a significant level of heterogeneity in the results, greater for specificity than for sensitivity (Fig. 5). The value for sensitivity was >80% in all the studies but one.^41^ The value for specificity was mainly >30%; however, in the remaining studies, the low specificity was accompanied by a very high sensitivity. However, because of important variation across studies with simultaneous presence of factors (time, presence of extracranial injuries, mixed populations) (Fig. S2) with potentially contrasting effects on the accuracy estimates and lack of individual data and/or insufficient number of studies, we were unable to compare patient characteristics and investigate the effect of the planned sources of heterogeneity (see online supplementary material at http://www.liebertpub.com). Poor reporting of patient and study information also contributed to unknown sources of heterogeneity.

One study was an outlier (Zongo and colleagues).^42^ Exclusion of this study made no change in sensitivity (96.3% vs 96.1%); however, specificity increased from 31% to 33%. This could be explained by the fact that in this study, including the greatest number of patients, S100B levels were measured in plasma, thus increasing the probability of false positive results (Fig. S3) (see online supplementary material at http://www.liebertpub.com).

To explore the effect of risk of bias in the patient selection domain on the summary estimates, we excluded eight studies considered at high (*n* = 1) or unclear (*n* = 7) risk of bias. The exclusion of these studies slightly improved sensitivity (98%) (Fig. S4) (see online supplementary material at http://www.liebertpub.com). A sensitivity analysis was also undertaken to assess the impact of studies containing mixed populations on our findings. We excluded one study (Welch and colleagues),^40^ because the authors included patients with moderate TBI (GCS 9–12). There was no impact on our findings. Four studies enrolled a mixed pediatric and adult population. Exclusion of these studies as well as those in which this information was unclearly reported made no difference to our results (Fig. S4).

The prevalence of CT findings was relatively high (> 11%) in seven studies. Excluding these studies resulted in a slight increase in sensitivity and a slight decrease in specificity (98% and 29%, respectively). Finally, eight studies considered skull fracture as a CT abnormality. To explore the impact of the type of reference standard on the summary estimates, we excluded these studies as well as those in which this information was unclearly reported. The exclusion of these studies slightly impacted sensitivity and specificity (93% and 35%, respectively) (Fig. S4).

### Quality of evidence of S100B

**FIG. 6.**
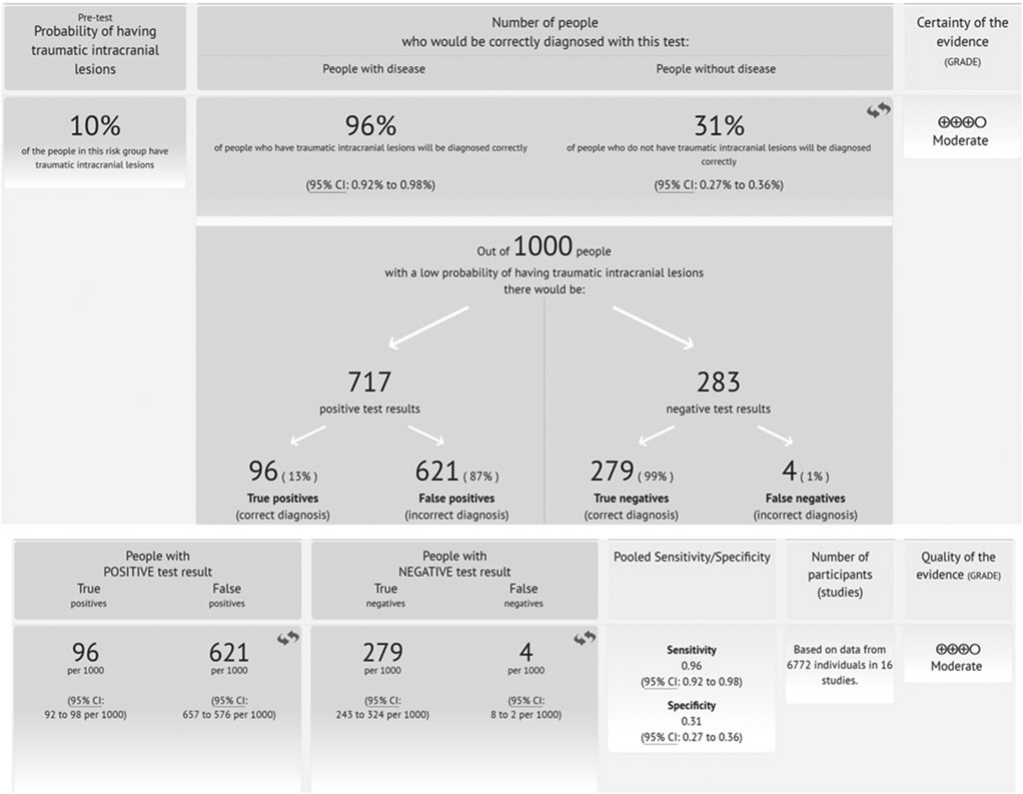
Summary of evidence for the use of blood S100 calcium binding protein B (S100B) protein concentrations (0.10–0.11μg/L cutoff) to diagnose brain injury as assessed by CT scan in patients with mild traumatic brain injury (mTBI).

The quality of the evidence for the use of blood S100B levels to diagnose brain injury as assessed by CT scan in patients with mild TBI was moderate (Fig. 6).

### GFAP

Eligible studies reporting the accuracy of GFAP for detecting intracranial lesions on CT scan comprised three cohorts with mild to moderate TBI patients and one cohort with mild to severe TBI patients (783 patients) (Figs. 2 and 3).^29,34,36,40^ All studies were recent publications (2012–2016).

The individual sensitivities were between 67% and 100%, whereas the specificities were between 0% and 89%. Sensitivities were sufficiently homogenous, whereas specificities were clearly heterogeneous. The thresholds used, ranging from 0 ng/mL^40^ to 0.6ng/mL^29^ were not pre-specified, and were determined from ROC analyses. The summary ROC curve of the accuracy of GFAP across all four studies, regardless of the threshold used, is shown in Figure 3.

The planned comparison between S100B and GFAP diagnostic performance was not possible, because of the limited number of studies and different spectrum of patients available for GFAP.

### NSE

The accuracy of NSE for discriminating between TBI patients with intracranial lesions on CT scanning from those without lesions was evaluated in three studies (314 patients).^33,41^ Figure 2 shows a forest plot of the individual study estimates of sensitivity and specificity. The sensitivities were between 56% and 100%, whereas the specificities were between 7% and 77%. The studies reported a considerable variation in the threshold adopted, ranging from 9 to 14.7 μg/L (Table 3).

### UCH-L1

The accuracy of the initial circulating UCH-L1 levels for detection of intracranial lesion on CT was evaluated in two very recent studies (96 and 251 patients respectively)^35,40^ including both mild to moderate adult TBI patients (GCS 9–15). The two studies yielded the same sensitivity of 100% (95% CI 88–100) and specificities of 21% (95% CI 12–32) and 39% (95% CI 33–46) (Fig. 2). They reported similar thresholds (0.029–0.04 ng/ml) and used the same assay (Table 3).

### Tau

The accuracy of circulating tau (cleaved tau [C-tau]) for diagnosis of CT abnormalities was evaluated only in one small study (50 patients).^28^ The sensitivity was 50%, whereas the specificity was 75%. Among the 10 patients with abnormal findings on CT enrolled in this study, 5 (50%) had no detectable C-tau levels.

## Discussion

In this systematic review, we have provided a comprehensive and thorough examination of the literature on protein biomarker diagnostic signatures for traumatic brain lesions to define how to best take advantage of these tests in ED daily patient care. We found that of the six biomarkers explored, current evidence only supports the measurement of S100B to help informed decision making in patients presenting to the ED with suspected intracranial lesion following mild TBI, possibly reducing resource use. There is as yet insufficient evidence that GFAP, NSE, and UCH-L1 are ready for clinical application, despite their unequivocal association with TBI. Further, tau and neurofilament proteins were analyzed in too few studies to draw any meaningful conclusions. Importantly, serious problems were observed in many of the studies, ranging from unfocused design and inappropriate target groups to biased reporting and inadequate analysis. These points are further elaborated in the subsequent discussions.

### S100B

Our findings demonstrate the clinical utility of S100B for the intended use of allowing physicians to be more selective in their use of CT without compromising care of patients with mTBI. More specifically, the 16 studies applying the same prespecified cutoff of 0.10–0.11μg/L yielded a pooled sensitivity of 96% (95% CI 92–98%) and specificities of 31% (95% CI 27–36%). Assuming a pre-test probability of 10%^44^ would mean that, overall, 100 of 1000 tested patients will have a final diagnosis of intracranial lesion. The pooled results obtained for sensitivity and specificity would mean that, of these, between 92 and 98 will test positive (true positives) and 2–8 will test negative (false negatives). Of the 900 with negative CT, between 243 and 324 will test negative (true negatives) and between 576 and 657 will test positive (false positives) (Fig. 6).

Even though this high sensitivity and excellent negative predictive value looks promising, information regarding which lesions could be missed and the associated consequences—if left untreated—is particularly relevant to the broad acceptance and adoption of S100B by the medical community. Accordingly, there is an ongoing debate about the risk of sending home a misdiagnosed patient with a potentially life-threatening condition such as an epidural hemorrhage. From the available data,^3,19,30,32,39,42^ we were unable to identify specific types of injury that were systematically missed, albeit subdural hematomas were slightly more frequently misclassified as false negatives. We speculate that this may be because of the brain lesion location and/or extension as well as the pathoanotomical and neurovascular features of the different injuries that cause an altered or delayed leakage of S100B into the circulation. Importantly, one study^30^ demonstrated that lesions requiring surgery (one subdural hematoma and one epidural hematoma) were missed by S100B, thereby indicating that this marker—if used alone as a diagnostic tool—is not completely reliable. Given that distinct patterns of injury are linked to patient-specific variability, efforts must to be made to develop advanced multiparameter-based solutions integrating marker signature and patient features. Such multimodal prediction models could be more suitable for an accurate diagnosis, characterization of injury types, and risk stratification of mTBI patients.^45^

It will be also critical to estimate the independent and complementary value of biomarkers and determine whether this strategy provides added diagnostic utility when combined with a careful clinical assessment or when integrated into existing clinical decision rules for the selective use of CT, such as the CT in Head Injury Patients (CHIP) model,^46^ the New Orleans criteria,^4^ or the Canadian Head CT rule.^47^ Unless a biomarker-based approach yields an incremental diagnostic value and clearly demonstrates its superiority over standard, readily available patient characteristics, the broad acceptance in medical practice is unlikely.^48^

Reliability and reproducibility of S100B results also requires a critical consideration of the comparability and potential variability in biomarker measurements when using assays from different manufacturers. We found the adoption of a relatively uniform and standardized approach for S100B determination, with 14 studies using the ECLIA Elecsys® Roche and 2 studies using the ILMA LIA-mat Sangtec 100. These two automated immunometric assays have been demonstrated to have a good correlation, with almost identical diagnostic capability,^27^ therefore excluding that this factor could have influenced our conclusions. A comparable level of consistency in analytical methods and assays used is not available for any of the other biomarkers considered in this review.

Our review showed that the results across S100B studies using the prespecified cutoff were consistent in terms of sensitivities and specificities, with only one outlier showing an exceptionally low specificity (12%).^42^ A plausible explanation for this anomaly is that in this study, plasma samples were used to measure S100B. This interpretation fits well with evidence from previous literature demonstrating how the interference of the anticoagulant on the immunoreactivity for S100B can alter its levels relative to serum (values higher by ∼20%).^49^ Consequently, in the study of Zongo and colleagues, the use of the prespecified cutoff for serum inevitably resulted in a systematic increase of false positive results.^42^ This observation, while complicating the analysis of S100B blood levels, points to the need for a more exhaustive knowledge and understanding of pre-analytical factors as potential confounders and sources of variability, and supports the adoption of different cutoff values, depending on the sample type used. Intriguingly, this observation suggests that plasma could be more suitable and possibly desirable for measuring S100B levels in mild TBI patients, because of very low concentrations in this population. However, even after removing the outlier, a considerable heterogeneity remained, necessitating caution when interpreting analysis results.

Investigations from multiple research groups provided evidence that a series of factors other than the brain injury may influence levels of biomarkers in the circulation and, therefore, the diagnostic accuracies. Such factors encompass biomarker characteristics such as molecular weight; injury-specific release mechanisms and clearance (Table S1);^50,51^ patient features including presence of extracranial injuries or polytrauma, intoxication, location of the injury, and even genetic, pre-analytical and laboratory-dependent procedures including all steps from management of equipment to execution of assays manufacturing processes; and post-analytical data handling.^19,52–54^ We were not able, however, to systematically investigate these potential sources of heterogeneity, because of a substantial variation across studies, the suboptimal reporting of patient and study information, and the coexistence in the same study of factors with contrasting or controversial effects on the accuracy estimates. Taken together, these findings demonstrate that future research must be refined by improvements in study design as well as standards and characterization of patient selection (See box on page 17) .

In this regard, surprisingly, we noted that to date no attempt has been made to specifically investigate the effect of comorbidities and sex on the diagnostic performance of S100B or any other marker. Sex is recognized as a primary determinant of biological variability, responsible for anatomical, neurochemical, and functional brain connectivity differences, heavily influencing neurobiological and neuropathophysiological response.^55^ It is also associated with important differences in hormones, metabolism, and the immunological system, which in turn may interfere with the determination of circulating TBI biomarker.^56^ Factoring sex into research designs and analyses is a theme under active debate, and is considered fundamental to rigorous and relevant biomedical research. Hence, we emphasize that this is a critical knowledge gap for future investigation, especially in light of the mounting evidence of the changing gender pattern caused by the shift in the TBI population toward older age, also at risk of multiple comorbid conditions (see Thaler and colleagues).^39^ Systematic reviews and meta-analyses of individual participant data (IPD) may represent a powerful approach to overcome some of these gaps and limitations,^57^ also supported by the current initiatives to share clinical data and the establishment of common repositories, such as the Federal Interagency Traumatic Brain Injury Research (FITBIR) database (https://fitbir.nih.gov/).^58^

Clinical application of S100B implies that choosing the right assessment time point (time between injury and sampling)^59^ is an integral part of the test. Based on the results of S100B kinetics studies, guidelines have specifically indicated a time window within 3^9,60^ to 6^9^ h post-injury for S100B to detect intracranial lesions. A recent study supported a 3 h window for safe rule-out of acute intracranial lesion in clinical practice, showing that a second blood sampling 3 h after the first one is not informative and resulted in a non-trivial loss of sensitivity of ∼6% (e.g., eight patients with positive CT would have been missed).^27^ We were unable to further address this specific issue in this review because of the heterogeneity in study design. In addition to post-injury delays in sampling, the delay from obtaining samples to processing and analysis, and the storage conditions during this delay could both be important modulators of S100B stability and assay results. Age, gender, and comorbidities or their combination can also importantly affect the kinetics of S100B.^61^ Future studies should inform whether these variables should be considered, and what the potential influence on biomarker results and interpretation is.

The results of our study expand and corroborate those from previous systematic reviews and meta-analyses,^62–64^ and confirm that the implementation of S100B might allow a reduction of the number of CT scans by ∼30%.^3^ These considerations also have broad financial implications for healthcare costs. However, none of the studies in our review explored the cost effectiveness of the use of biomarkers, and the few economic studies and data in the literature are controversial. An earlier study by Ruan and colleagues^65^ reported a limited effect of S100B on healthcare resources and a potential economic impact only in specific clinical scenarios (i.e., CT scanning rate >78% or a faster turnaround time of biomarker results of at least 96 min compared with CT scan results). Conversely, in a more recent cost analysis conducted in a Swedish regional hospital, the clinical use of S100B incorporated into the Scandinavian guidelines substantially reduced healthcare costs, especially in cases of strict adherence to management recommendations (71€ per patient).^66^ These results are not generalizable, and must be carefully interpreted according to their specific contexts, because of the differences across countries, healthcare systems, hospital settings, and ensuing care patterns. To refine cost calculations, future studies should take these factors into consideration, as well as CT overutilization and the socioeconomic costs associated with increased cancer risks from CT scans. Clear demonstration of cost saving and added benefits beyond those obtained by current management strategies for mTBI are essential for TBI biomarkers to be adopted and widely used by the medical community.

### GFAP

Recent narrative reviews have outlined the potential of GFAP for identifying patients with intracranial lesions after head trauma,^7^ but none of these used systematic review methods or meta-analyses. In the meta-analysis reported here, we included four studies, in which the diagnostic accuracy of GFAP reflected sensitivities of 67^29^–100%^36,40^ and specificities of 0^40^–100%.^29^ Although promising, these results must be approached with caution, because the studies included patients with severe and moderate TBI not representative of the target population of the test (the median prevalence of abnormal CT findings across the studies was 22%), and thresholds were not prespecified, factors that may have inflated the accuracy estimates.^67^ For diagnostic validation, it will be fundamental to establish reliable and valid thresholds. Also, GFAP needs be tested in larger clinical studies with a focus on the *intended use*.^68,69^ To this end, it has been argued that studies investigating the implementation of biomarker measurements in guidelines for mTBI management—to avoid use of unnecessary CT—should be limited to patients currently recommended for such examination (GCS 14–15), therefore excluding patients with GCS score of 13 for whom biomarker assessment would not add to clinical examination.^9^ As mentioned earlier, the definition of these setting-specific characteristics is also critical for performing reliable cost analyses and determining the primary economic advantage of using blood biomarkers as a pre-head CT screening tool.

A meaningful comparison between GFAP and S100B diagnostic performances was precluded by a substantial difference in study populations. In this context, we note that TBI biomarkers discussed in this review are usually considered individually. Further work should more consistently explore simultaneous assessment of multiple biomarkers providing the framework for comparing the accuracy of tests that have directly been compared in individual studies.

### NSE and UCH-L1

The relative dearth of studies evaluating the diagnostic accuracy of NSE, UCH-L1, and Tau in the ED for identifying patients with intracranial lesions following mTBI hampered the possibility of performing meta-analyses. The diagnostic value of NSE remains uncertain, with studies showing remarkable variations and inconsistency. In contrast, the accuracy of UCH-L1 for detecting intracranial lesions on CT scan was evaluated in two studies that yielded an optimal sensitivity (100%) but modest specificities (21–39%). Similar to GFAP, the thresholds used were not prespecified, and the studies included patients with mild to moderate TBI (GCS 9–15). Hence, further studies are required to confirm the reproducibility of these findings and to determine clinical utility in daily bedside care.

### Tau and neurofilament proteins

There is insufficient evidence to support the clinical validity of initial circulating c-Tau or neurofilament protein concentrations for the management of patients with mTBI.

### Implications for research and practice: Strengths and weakness of the review

Our current insight appreciates the complexity of the pathobiology of TBI most probably requiring multifaceted, multimodal approaches, integrating biomarkers and traditional clinical characteristics to allow a more powerful and accurate characterization and risk stratification of mTBI,^45,70^ a premise currently insufficiently reflected in the literature. In addition, if the different biomarkers do indeed reflect different pathophysiological processes^51^ with independent information about imaging abnormality, outcome impact, and different diagnostic windows, it is possible that the use of a panel of biomarkers may substantially increase the diagnostic specificity for the end-point of interest.^71,72^ Unfortunately, to date, only a few such studies are available. More data are needed to evaluate whether a multi-marker approach based on a panel of biomarkers with distinct time-dependent discriminatory accuracy provides a better performance for the detection and characterization of TBI.

Further, we should be cautious in using CT as a gold standard to judge the performance of circulating biomarkers. When compared with MRI, there is increasing recognition that X-ray CT provides poor sensitivity for structural lesions in TBI such as microbleeds and diffuse axonal injury.^73,74^ It follows that we cannot assume that false positivity in detection of CT-visible abnormality equates to false positivity in detection of structural injury, because some of these false positives may be associated with abnormalities on MRI or other advanced neuroimaging, persistent post-concussive symptoms, or long-term neurological, cognitive, and/or neuropsychiatric complications.^75–78^ On the other hand, these considerations suggest a broader clinical application of a biomarker-based strategy for diagnosis and management of mTBI. Biomarkers could be used to provide guidance for prognostic groupings, to refine risk stratification, and to inform and guide different management and treatment decisions including indications for advanced MRI techniques (diffusion tensor imaging [DTI], susceptibility weighted imaging [SWI], functional connectivity MRI [fcMRI]), enrollment into clinical trials, and closer monitoring and follow-up of mTBI patients.

From a clinical perspective, biomarkers are not useful if they do not provide real-time decision support for diagnosis of mTBI at the bedside in the ED. A successful approach to the rapid incorporation into routine patient care will be to develop an automated multiplex point of care (POC) device, capable of providing accurate measurements to the clinician at a reasonable cost and with short turnaround times (∼15–20 min).^52,53^

The studies discussed in this review focus primarily on adult patients. There is, however, a growing interest in using biomarkers to optimize diagnosis and management of pediatric mTBI, because of the high risk of TBI in children ≤4 years of age, the difficult functional assessments, and the radiation exposure at a young age with ensuing increased cancer risk.^75,79,80^ Future studies and systematic reviews taking current and new evidence into account are urgently needed to elucidate the role of biomarkers and establish their clinical utility in this special and vulnerable population.

Several potential limitations merit consideration. Patient selection is a critical aspect in reviews of test accuracy, as it can alter the spectrum of disease and non-disease and the prevalence in the population, strongly impacting test accuracy.^67^ Given the heterogeneous and polymorphous nature of TBI, in particular at the milder end of the spectrum, there has been an inconsistent, sometime controversial, definition of mTBI adopted in the included studies. For example, focal neurological deficit has been considered either as an inclusion or as an exclusion criterion (Table S2). This diagnostic uncertainty may possibly have introduced different biases. Although this is an issue that we cannot solve in this review as we had to rely on the criteria that were listed in the included studies; nonetheless, we were able to assess the robustness of the findings using sensitivity analysis, which even demonstrated an improvement in S100B performance (Fig. S4).

However, with respect to selection of patients and study design, our group endorses the importance of methodological rigor, and advocates the use of standardized protocols and a prespecified set of data analysis both as a means to reduce related biases and inadequate reporting, and as a mandatory prerequisite to ensure successful validation and implementation of TBI diagnostic biomarkers. Also critical consideration for sample size planning based on assay precision, clinical significance, and regulatory considerations is necessary. Involvement of regulatory bodies in driving forward harmonization and standardization is considered essential. A major step forward in this direction is the recently established collaboration between researchers and the United States Food and Drug Administration (FDA) in the context of the TBI Endpoints Development (https://tbiendpoints.ucsf.edu/).

Further, despite the broad adoption by the scientific community of the STARD statement (Standards for Reporting of Diagnostic Accuracy studies),^81^ we found a number of studies with poor or inconsistent reporting of important information, including patient and specimen characteristics, assay methods, handling of missing data, and statistical analysis methods, in addition to suboptimal descriptions of study findings, which hampered our assessment of potential for bias and interpretation of the results. Our observations are important in raising awareness of key reporting issues in many of the TBI diagnostic studies. The STARDdem Initiative recently proposed an implementation of the STARD statement with guidance pertinent to studies of cognitive disorders, which is expected to contribute to the development of Alzheimer biomarkers.^82^ A similar initiative for TBI biomarker studies could increase transparency and the quality of information provided by such studies, enabling evaluation of internal and external validity and, consequently, a more effective translation and application of their findings to clinical practice.

Harmonization and standardization of biomarker assays that can reliably quantify biomarkers with high analytical precision is critical to ensure that measurements are reproducible and consistent across different analytical platforms and multiple laboratories.

## Conclusion

Based on this review, we found that measurement of S100B can help informed decision making in the ED with respect to the selection of adults with a mTBI for CT scan, possibly safely reducing resource use. Conversely, there is little evidence for clinical application of GFAP, UCH-L1, NSE, tau or neurofilaments. However, much work remains to evaluate factors that may influence biomarker levels, and a critical confrontation is required with the implications for actual management, clinical impact, and health economic implications. We also found serious problems in the design, reporting, and analysis of many of the studies, emphasizing the importance for the research community to establish methodological standards and acquire extensive high-quality data for TBI biomarker validation. This is an essential prerequisite for drawing firm conclusions about the performance of tests based on these biomarkers and their clinical utility.

Finally, through the extensive and critical review of the current TBI biomarker existing literature, and state-of-the-science discussions with key opinion leaders and subject matter experts, members of our work group collaborated to evaluate the evidence necessary to demonstrate clinical utility of TBI biomarkers, to identify critical gaps for advancing the field, and to lay the foundation for a “*living*” TBI biomarker registry capable of providing an up-to-date list and information on biomarker studies and their results (see Box). Such a strategy, helping to foster collaboration, developing the high levels of evidence needed to support analytical validity and clinical utility, and improving the quality of assessments of novel candidate biomarkers, should establish the solid ground needed for changing biomarker research from data that informs into data that transforms, turning knowledge into a new medical practice.

## Supplementary Material

Supplemental data

## Appendix: Search Strategy

### MEDLINE^®^ (Ovid) 1946 to October Week 2 2016

1. brain injuries/
2. craniocerebral trauma/
3. head*.ti,ab.
4. brain*.ti,ab.
5. injur*.ti,ab.
6. trauma*.ti,ab.
7. 3 or 4
8. 5 or 6
9. 7 and 8
10. or/1-2,9
11. biological markers/
12. biomarker.ti,ab.
13. marker*.ti,ab.
14. biomarker*.ti,ab.
15. or/11-14
16. S-100*.ti,ab.
17. S100*.ti,ab.
18. S100 proteins.ti,ab.
19. S100 Proteins/
20. or/16-19
21. GFAP.ti,ab.
22. glial protein*.ti,ab.
23. glial fibrillary acidic protein*.ti,ab.
24. glial intermediate filament protein*.ti,ab.
25. astroprotein*.ti,ab.
26. GFA-protein*.ti,ab.
27. glial fibrillary acidic protein/
28. or/21-27
29. C-tau.ti,ab.
30. cleaved-tau.ti,ab.
31. tau protein*.ti,ab.
32. p-tau.ti,ab.
33. tau proteins/
34. or/29-33
35. NSE.ti,ab.
36. neuron specific enolase*.ti,ab.
37. gamma-enolase*.ti,ab.
38. enolase 2.ti,ab.
39. nervous system specific enolase*.ti,ab.
40. phosphopyruvate hydratase*.ti,ab.
41. phosphopyruvate hydratase/
42. or/35-41
43. UCH-L1.ti,ab.
44. UCHL1.ti,ab.
45. ubiquitin carboxyl-terminal hydrolase L-1.ti,ab.
46. ubiquitin c-terminal hydrolase*.ti,ab.
47. ubiquitin carboxy- terminal esterase*.ti,ab.
48. ubiquitin thiolesterase*.ti,ab.
49. ubiquitin carboxyl-terminal hydrolase L-1, human.ti,ab.
50. UCHL1 protein.ti,ab.
51. ubiquitin/
52. ubiquitin thiolesterase/
53. or/43-52
54. NF-H.ti,ab.
55. NFH.ti,ab.
56. NFP-200.ti,ab.
57. NFP200.ti,ab.
58. hyperphosphorylated neurofilament*.ti,ab.
59. neurofilament protein*.ti,ab.
60. neurofilament H protein*.ti,ab.
61. neurofilament triplet protein*.ti,ab.
62. neurofilament protein H.ti,ab.
63. phosphorylated neurofilament.ti,ab.
64. neurofilament proteins/
65. or/54-64
66. blood.ti,ab.
67. serum.ti,ab.
68. plasma.ti,ab.
69. or/66-68
70. or/15,20,28,34,42,53,65
71. and/10,69-70
72. 71 not (animals/ not humans.sh.)

### Embase (OVID) 1980 to 2016 Week 43

1. exp brain injury/
2. craniocerebral trauma/
3. (head* and injur*).ti,ab.
4. (brain* and injur*).ti,ab.
5. ((head* or brain*) and trauma*).ti,ab.
6. or/1-5
7. exp biological marker/
8. biomarker.ti,ab.
9. (marker* or biomarker*).ti,ab.
10. or/7-9
11. (blood or serum or plasma).ti,ab.
12. exp blood/
13. exp serum/
14. exp plasma/
15. or/11-14
16. exp prognosis/
17. prognos*.ti,ab.
18. exp diagnostic procedure/
19. diagnos*.ti,ab.
20. di.fs.
21. or/16-20
22. and/6,10,15,21
23. animal/ not human/
24. 22 not 23

### Cochrane Library (searched 19 October 2016)

#1 MeSH descriptor: [brain injuries] explode all trees

#2 MeSH descriptor: [craniocerebral trauma] explode all trees

#3 (head* or brain*) and (injur* or trauma*):ti,ab,kw (word variations have been searched)

#4 (#1 OR #2 OR #3)

#5 MeSH descriptor: [biomarkers] explode all trees

#6 (biomarker* or marker*):ti,ab,kw (word variations have been searched)

#7 (#5 OR #6)

#8 MeSH descriptor: [blood] explode all trees

#9 MeSH descriptor: [serum] explode all trees

#10 MeSH descriptor: [plasma] explode all trees

#11 (blood OR serum OR plasma):ti,ab,kw (word variations have been searched)

#12 (#8 OR #9 OR #10 OR #11)

#13 (#4 AND #7 AND #12)

## Notes

Panel: Consensus-Based Recommendations to Enhance Adoption of Diagnostic Traumatic Brain Injury Biomarkers in the Clinic 1. Standardized Study and Analysis Protocols and Methodological Rigor

▪ Focus on “real-world” clinical questions (appropriate target populations) to optimize clinical translation effectiveness and measure of healthcare economic implication.
▪ Increase transparency and quality of reporting by calling on investigators to adopt optimal/consolidated guidelines for reporting biomarker work (http://www.stard-statement.org/).
▪ Reduce biases by implementing critical appraisal tools for evaluating the quality of research (http://www.quadas.org/).
▪ Develop internationally accepted common reference standards and reference methods to reduce the variability while permitting reliability of biomarker results, reproducibility, and comparability across analytical platforms/laboratories and clinical studies, and the establishment of general exact diagnostic cutoffs.

2. Additional Knowledge Needed to Improve Reliability in the Use of Blood Biomarkers and to Ensure a Successful Validation and Implementation in Clinical Practice

▪ Assess relationships between specific types and patterns of injury and biomarker kinetics.
▪ Factor primary biological and clinical variables, including sex and comorbidities, into research design and analyses to exhaustively understand their influence on biomarker pathophysiology and levels.
▪ Separate and systematically explore special populations (e.g., geriatric and pediatric traumatic brain injury [TBI]).
▪ Take a thorough investigative approach accounting for pre-analytical factors and adoption of different cutoff values and alternative/complementary time points.

3. Exploration of Novel Opportunities and Strategies for Expanding and Informing Biomarker Clinical Research as a Basis for Developing Multimodal Multidimensional Models to Diagnose Mild TBI

▪ Simultaneous assessment of multiple biomarkers to compare accuracy and evaluate the performance of multi-marker panels for the detection and characterization of TBI.
▪ Sharing of clinical data and establishment of common repositories to support individual participant data meta-analyses (IPD-MAs) for more robust development of diagnostic models tailored to specific (sub)populations or settings, and testing their generalizability and usefulness.
▪ Systematic and rigorous evaluation, quantification, and demonstration of the incremental diagnostic value of TBI biomarkers over standard, readily available patient characteristics, and existing prediction rules for the selective use of CT.
▪ Combination of brain injury biomarkers and patient characteristics yielding independent and incremental diagnostic information toward a powerful multi-parameter platform to assist and enhance clinical decision making (triage for CT scanning) in patients with mTBI at the bedside in the emergency department.
