# Supplementary material for: Blood-Based Protein Biomarkers for the Management of Traumatic Brain Injuries in Adults Presenting to Emergency Departments with Mild Brain Injury: A Living Systematic Review and Meta-Analysis
Source: J Neurotrauma. 2021 Apr 5;38(8):1086–106. doi: 10.1089/neu.2017.5182 (PMC8054517; doi:10.1089/neu.2017.5182)
Supplement: Supplemental data [file Supp_Data.pdf]

Supplementary Data

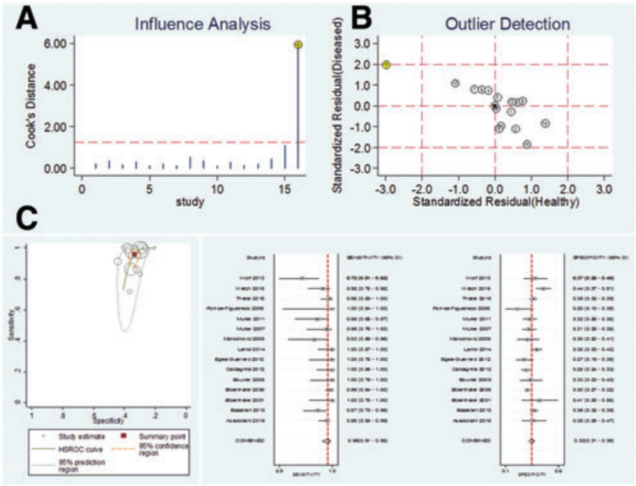

SUPPLEMENTARY FIG. S1. Graphical depiction of **(A)** influence and **(B)** outlier detection analyses of S100 calcium binding protein B (S100B) 0.10–0.11 μg/L cutoff value studies. **(C)** Summary receiver operating characteristics plot of sensitivity and specificity of S100B at 0.10–0.11 μg/L cutoff value after removing the influential study (Zongo and colleagues[42]).

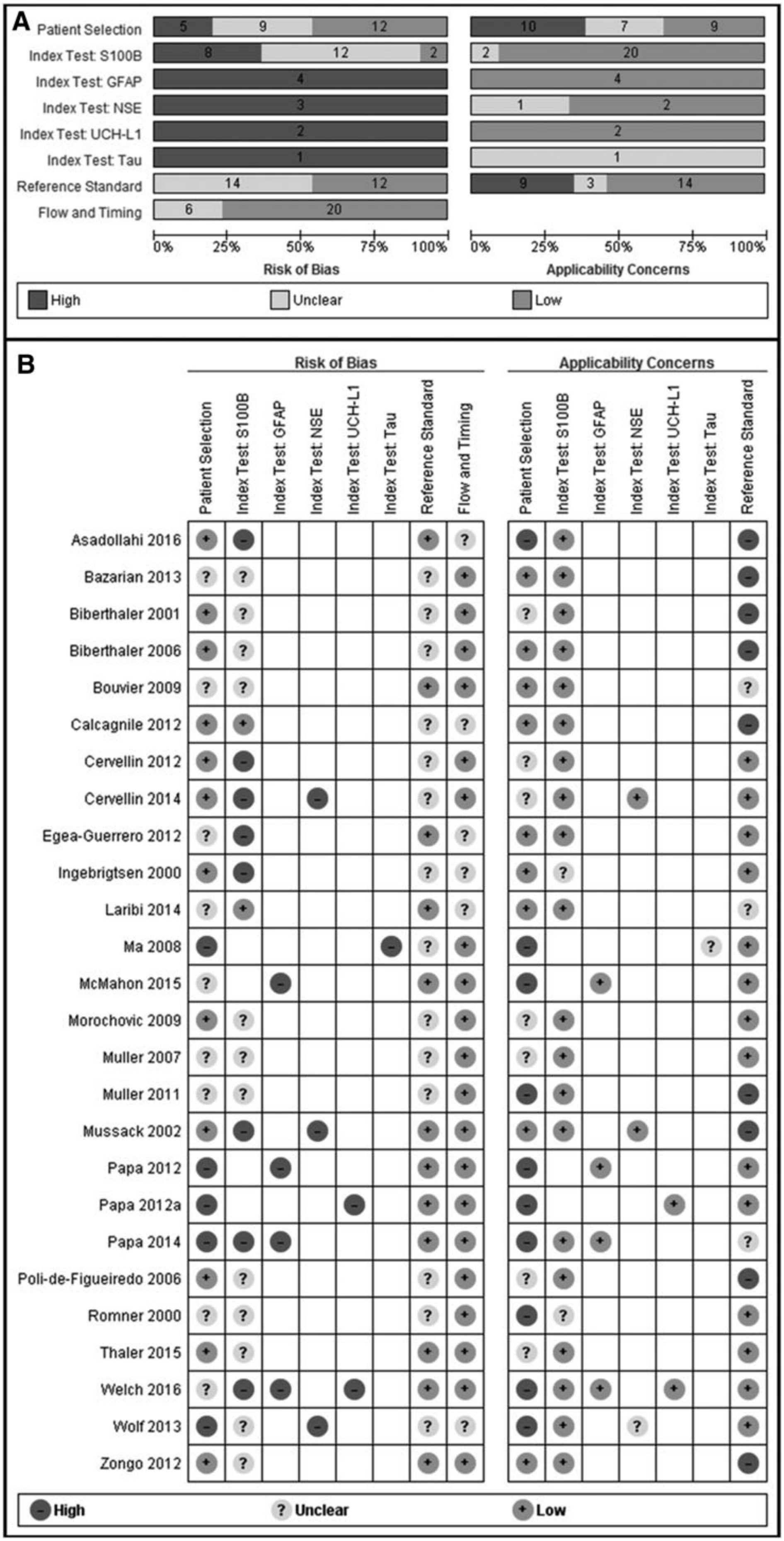

SUPPLEMENTARY FIG. S2. **(A)** Risk of bias and applicability concerns graph by marker. Review authors' judgments about each domain presented as percentages across included studies. **(B)** Risk of bias and applicability concerns summary by marker. Review authors' judgments about each domain for each included study.

Study	TP	FP	FN	TN	Sampling Time	Polytrauma	CT abnormalities	GCS	Population Age	Sensitivity (95% CI)	Specificity (95% CI)
Asadollahi 2016	75	51	4	28	Within 3 h	No	Skull fracture included	13-15	Adult	0.95 [0.88, 0.99]	0.35 [0.25, 0.47]
Bazarian 2013	39	473	6	264	Within 6 h	Yes	Skull fracture included	13-15	Adult&Pediatric	0.87 [0.73, 0.95]	0.36 [0.32, 0.39]
Biberthaler 2001	15	22	0	15	Within 3 h	No	Skull fracture included	13-15	Unclear	1.00 [0.78, 1.00]	0.41 [0.25, 0.58]
Biberthaler 2006	92	855	1	361	Within 3 h	No	Skull fracture included	13-15	Adult	0.99 [0.94, 1.00]	0.30 [0.27, 0.32]
Bouvier 2009	16	60	0	29	Within 3 h	No	Unclear	13-15	Adult	1.00 [0.79, 1.00]	0.33 [0.23, 0.43]
Calcagnile 2012	24	350	0	138	Within 3 h	Unclear	Skull fracture included	14-15	Adult	1.00 [0.86, 1.00]	0.28 [0.24, 0.33]
Egea-Guerrero 2012	15	94	0	34	Within 6 h	Yes	Skull fracture excluded	15	Adult&Pediatric	1.00 [0.78, 1.00]	0.27 [0.19, 0.35]
Laribi 2014	26	231	0	143	Within 3 h	No	Unclear	13-15	Adult	1.00 [0.87, 1.00]	0.38 [0.33, 0.43]
Morochovic 2009	15	59	3	25	Within 3 h	Yes	Skull fracture excluded	13-15	Adult	0.83 [0.59, 0.96]	0.30 [0.20, 0.41]
Muller 2007	20	141	1	64	Within 12 h	No	Skull fracture excluded	13-15	Adult	0.95 [0.76, 1.00]	0.31 [0.25, 0.38]
Muller 2011	19	144	3	67	On admission	No	Skull fracture included	13-15	Adult&Pediatric	0.86 [0.65, 0.97]	0.32 [0.26, 0.38]
Poli-de-Figueiredo 2006	6	35	0	9	Within 3 h	No	Skull fracture included	13-15	Unclear	1.00 [0.54, 1.00]	0.20 [0.10, 0.35]
Thaler 2015	49	474	1	258	Within 3 h	No	Skull fracture excluded	13-15	Adult	0.98 [0.89, 1.00]	0.35 [0.32, 0.39]
Welch 2016	33	120	3	95	Within 6 h	Unclear	Skull fracture excluded	9-15	Adult	0.92 [0.78, 0.98]	0.44 [0.37, 0.51]
Wolf 2013	18	52	7	30	Within 3 h	No	Skull fracture excluded	13-15	Adult	0.72 [0.51, 0.88]	0.37 [0.26, 0.48]
Zongo 2012	110	1272	1	177	Within 6 h	No	Skull fracture included	13-15	Adult&Pediatric	0.99 [0.95, 1.00]	0.12 [0.11, 0.14]

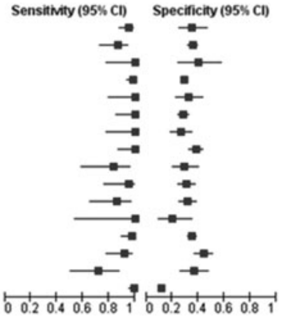

SUPPLEMENTARY FIG. S3. Forest plot of S100 calcium binding protein B (S100B) 0.10–0.11μg/L cutoff value studies for detection of CT abnormalities. Information related to population, sampling time and reference test—potential sources of heterogeneity—are shown.

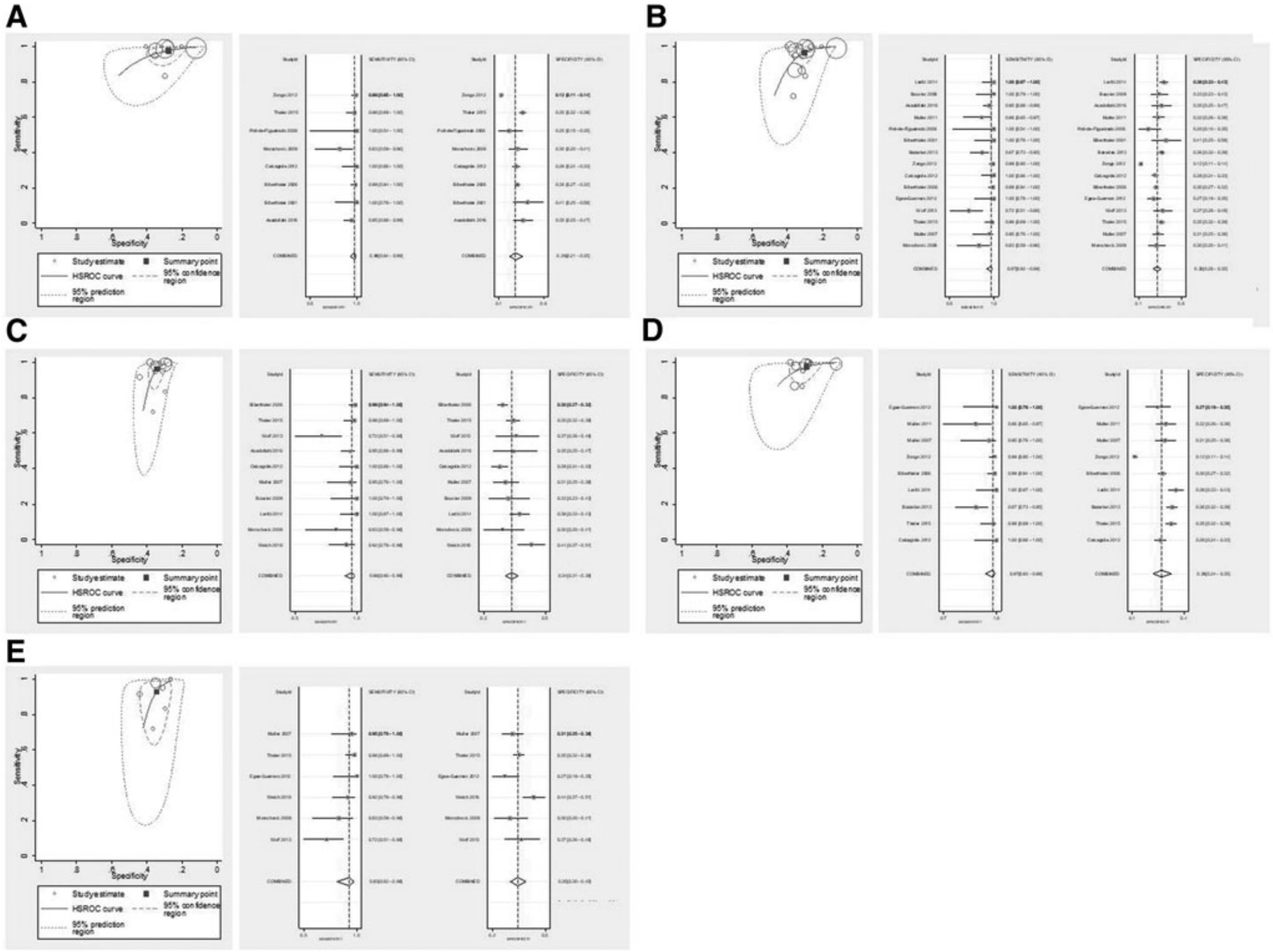

SUPPLEMENTARY FIG. S4. Detection of abnormal CT sensitivity analyses. **(A)** Detection of abnormal CT sensitivity analyses excluding studies at high or unclear risk of bias for patient selection domain. **(B)** Detection of abnormal CT sensitivity analyses excluding Welch and colleagues.[40] **(C)** Detection of abnormal CT sensitivity analyses excluding studies containing mixed pediatric and adult populations. **(D)** Detection of abnormal CT sensitivity analyses excluding studies having a high prevalence of CT findings (> 11%). **(E)** Detection of abnormal CT sensitivity analyses excluding studies at high or unclear risk of not properly classifying the target condition.

Supplementary Table S1. Summary of the Characteristics of the Protein Biomarkers Evaluated

Biomarker	*Location/ Protein attributes*	*Function/ Pathogenic process*	*Comment*	*Release kinetics in serum*
Glial markers				
S100B	Astroglial cells<br>MW 21 kDa bb homodimer	• Calcium-binding protein<br>• Involved in signal transduction and regulation of cell morphology, with neurotrophic properties	• Release from extracerebral tissues<br>• 100% renal clearance; levels potentially affected by renal insufficiency<br>• High normative concentrations in young children	Short half-life (~90–120 min)<br>Peak <6 h after injury
GFAP	Major constituent of glial filaments in astrocytes<br>Highly stable<br>MW 49.8 kDa	Cytoskeleton support	• Almost exclusively found in glial, negligible contribution of non-CNS-derived protein; raised expression in response to stimuli and injury;“ reactive astrogliosis ” after TBI<br>• Pathology-dependent generation of breakdown products (BDPs) of 38, 25, 20, and 18 kDa<br>• Presence of anti-GFAP auto-antibodies	The exact half-life is not yet understood<br>Peak at ~16–24 h after injury<br>Rapid appearance in serum post-injury, with levels detectable within 1 h
Neuronal and axonal markers				
NSE/γ–enolase	Prominently in the cytoplasm of neurons<br>Two γ-subunits ($\gamma\gamma$) MW 78 kDa	Involved with regulating intraneuronal chloride levels during neural activity	• Present in erythrocytes and platelets<br>• Hemolysis increases blood levels representing a significant artifact and source of error	Biological half-life of 48 h
UCH-L1	Neuronal cell body (perikarya)<br>Globular shape<br>MW 24 kDa	Protein de-ubiquitinization, playing a critical role in removal of damaged, misfolded, or overexpressed proteins both under normal and pathological conditions	• Resistant to degradation<br>• Implicated in familial Parkinsonism<br>• Elevated serum levels have been associated with abnormal BBB function after TBI<br>• High normative concentrations in young children and elderly subjects (≥ 65 years)	Peak ~8 h after injury<br>Rapid appearance in serum (< 1 h post-injury) with rapid decrease following TBI
Tau	Highly enriched in thin, nonmyelinated axons of cortical interneurons<br>Six isoforms<br>MW 48–68 kDa	Involved with assembling axonal microtubule bundles and participating in anterograde axoplasmic transport	• Possibly indicative of axonal damage in gray matter neurons<br>• Upon cellular injury, proteolytically cleaved into fragments of 10–18 kDa and 30–50 kDa (c-tau)<br>• Not completely specific for the CNS<br>• Injuries lead to the phosphorylation of tau, which can aggregate (tau tangles)	Levels peak 4–10 days after injury
Neurofilaments (NF)	Predominantly in axons<br>Heteropolymeric components<br>Three main subunits:<br>• Light (NF-L) MW 68–70 kDa<br>• Medium (NF-M) MW 145–160 kDa<br>• Heavy (NF-H) MW 200–220 kDa	Main component of the axonal cytoskeleton providing structural support and regulating axon diameter	• Potential specific measure of axonal injury<br>• Subcategory (Type IV) of intermediate filaments<br>• Phosphorylated heavy-chain neurofilament (pNF-H) is the extensively phosphorylated, axon-specific form of the NF-H subunit of the neurofilament and represents one of the most abundantly distributed axonal proteins	Unlike other markers, NF-L and pNF-H tend to continuously increase over time during the first 1–2 weeks and remain elevated 1 year after injury

BBB, blood–brain barrier; CNS, central nervous system; GFAP, glial fibrillary acidic protein; MW, molecular weight; NSE, neuron specific enolase; S100B, S100 calcium binding protein B; TBI, traumatic brain injury; UCH-L1, ubiquitin C-terminal hydrolase-L1.

Supplementary Table S2. mTBI and CT Abnormality Definition Used in the 26 Included Studies

Study ID	*Patients/Population definition*	*Reference standard (CT abnormality) definition*	*Skull fractures considered a CT abnormality*
Asadollahi 2016[18]	GCS score 13–15, LOC <30' and PTA <1h; exclusion criterion: focal neurological deficit	EDH, SDH, SAH, ICH, cerebral contusion, brain edema, depressed skull fracture	Yes, depressed skull fracture
Bazarian 2013[19]	CDC and prevention's definition: a blow to the head or rapid acceleration/deceleration resulting in at least one of the following: LOC <30', PTA <24 h, neuropsychological abnormality (any transient period of confusion, disorientation, or impaired consciousness; in children <2 yrs: irritability, lethargy, or vomiting post-injury), or neurological abnormality (seizure acutely after injury, hemiplegia, or diplopia). GCS score of ≥13 within 30' of the injury.	EDH, SDH, SAH, edema, skull fracture, and cerebral contusions.	Yes, skull fracture
Biberthaler 2001[20]	GCS score 13–15 at admission, and at least one of the following symptoms: amnesia, LOC, nausea, vomiting, vertigo, or severe headache. Exclusion criterion: focal neurological deficit	Hemorrhage, diffuse brain swelling, skull fracture	Yes, skull fracture
Biberthaler 2006 [3]	History of isolated head trauma; GCS score of 13–15 on admission; and one or more of 10 clinical risk factors: brief LOC, PTA, nausea, vomiting, severe headache, dizziness, vertigo, intoxication, anticoagulation, and age >60 yrs	Hemorrhage: EDH, SDH, SAH, ICH, ventricular, cerebellar, brainstem/ cortex contusion: hemorrhagic, non-hemorrhagic /fractures: skull cap, skull base, mastoid/ICP; focal and generalized brain edema	Yes, skull cap, skull base, mastoid
Bouvier 2009[21]	History of isolated head trauma; GCS score of 13–15 on admission; and at least one of the following symptoms: headache, nausea, vomiting, amnesia, LOC, focal neurological deficit, seizure, intoxication, age >60 yrs, anticoagulation, clinical signs of skull cap or skull base fractures.	EDH, SDH, SAH, skull fracture, cerebral contusions, petechial hemorrhage, pneumocephalus	Unclear
Calcagnile 2012[22]	History of head trauma. GCS 14–15 during examination and LOC <5' and/or amnesia. Subjects with neurological deficits and additional risk factors from the SNC guidelines (therapeutic anticoagulation or hemophilia, clinical signs of depressed skull fracture or skull base fracture, posttraumatic seizures, shunt-treated hydrocephalus, and multiple injuries) were excluded.	Any signs of cranial (skull fracture) or intracranial pathology (hematoma, air, or contusion)	Yes, skull fracture
Cervellin 2012[23]	MHI requiring CT scanning according to the local guideline:<br>GCS 14–15; history: LOS or PTA associated with at least one of the following: previous neurosurgical procedures, inherited coagulopathy or anticoagulant therapy, vomiting (more than 1 episode), epilepsy or post-traumatic seizures, worsening headache; clinical findings: drug or alcohol intoxication (even suspected), clinical signs of depressed or basilar skull fracture, focal neurological deficits	EDH, SDH, SAH, ICH, cerebral contusion, brain swelling	No
Cervellin 2014[24]	MHI, GCS 14–15 on admission; exclusion criteria: clinical signs of depressed or basilar skull fracture, focal neurological deficits.	EDH, SDH, SAH, ICH, cerebral contusion, brain swelling (brain edema)	No
Egea-Guerrero 2012[25]	GCS 15 at hospital admission and one or more of the following symptoms: transitory LOC; amnesia; persistent headache; nausea or vomiting; and vertigo	EDH, SDH, SAH, cerebral contusion	No

(*continued*)

Supplementary Table S2. (Continued)

Study ID	*Patients/Population definition*	*Reference standard (CT abnormality) definition*	*Skull fractures considered a CT abnormality*
Ingebrigtsen 2000[26]	Brain injury with brief (<10') LOC; GCS 13–15 at admission. LOC was considered to have occurred also when the patient had amnesia for the trauma.	Brain contusion, EDH, SAH	No
Laribi 2014[27]	MHI: GCS 13–15 with one or more of the following risk factors: amnesia, LOC, nausea, vomiting, vertigo, anticoagulation before injury, or severe headache on admission.<br>Exclusion criteria: focal neurological deficit, LOC >10'	NR	Unclear
Ma 2008[28]	mTBI: LOC and/or PTA, and admitted within 12 h of trauma with a GCS score 13–15. Exclusion criteria: focal neurological deficit, penetrating injury to the skull	EDH, SDH, SAH, ICH, cerebral contusion, hemorrhagic shear injury, intraventricular hemorrhage, pneumocephalus	No
McMahon 2015[29]	Positive clinical screen for acute TBI necessitating a noncontrast head CT according to ACEP/CDC evidence-based joint practice guidelines	Recommendations of the TBICDE Neuroimaging WG: cisternal effacement, mid-line shift, EDH, SAH, and intraventricular hemorrhage	No
Morochovic 2009[30]	mTBI: categories 1–3 according to EFNS<br>Category 1: GCS = 15, LOC <30 min, PTA <1 h, no risk factors<br>Category 2: GCS = 15 and risk factors present<br>Category 3: GCS = 13–14, LOC <30 min, PTA <1 h, with/without risk factors<br>Risk factors: unclear or ambiguous accident history, continued post-traumatic amnesia, retrograde amnesia >30 min, trauma above the clavicles, severe headache, vomiting, focal neurological deficit, seizure, coagulation disorder, high energy accident, intoxication with alcohol/drugs.	Acute EDH, SDH, SAH, parenchymal hematoma, cerebral contusion and brain swelling	No
Muller 2007[31]	History of brain injury, LOC or retrograde amnesia, GCS 13–15 on admission. Exclusion criterion: focal neurological deficit.	Brain contusion, SDH, EDH	No
Muller 2011[32]	Mild head trauma, GCS 13–15 on admission	Localized EDH, SDH, isolated SAH, skull fracture	Yes, skull fracture
Mussack 2002[33]	History of trauma, GCS 13–15 and at least one of the following symptoms: transient LOC (< 5 min), antero- or retrograde amnesia, nausea, vomiting, or vertigo	EDH, SDH, SAH, intracerebral hemorrhage, diffuse brain edema, skull fracture	Yes, skull fracture
Papa 2012[34]	Suspected mTBI: history of blunt head trauma followed by LOC, amnesia, or disorientation; GCS 9–15.	EDH, SDH, SAH, contusion, intracerebral hemorrhage, pneumocephalus, combination of lesions	No
Papa 2012[35]	Suspected mTBI: history of blunt head trauma followed by LOC, amnesia, or disorientation; GCS 9–15.	EDH, SDH, SAH, contusion, intracerebral hemorrhage, pneumocephalus, combination of lesions	No
Papa 2014[36]	Suspected mild or moderate TBI (mmTBI): history of blunt head trauma followed by LOC, amnesia, or disorientation; GCS 9–15.	NR	Unclear

(*continued*)

Supplementary Table S2. (Continued)

Study ID	*Patients/Population definition*	*Reference standard (CT abnormality) definition*	*Skull fractures considered a CT abnormality*
Poli-de-Figueiredo 2006[37]	Isolated MHI: GCS 13–15 and at least one of the following symptoms: amnesia, LOC, nausea, vomiting, vertigo, or severe headache. Exclusion criterion: focal neurological deficit.	Intracranial hemorrhage, skull fracture, and/or diffuse brain swelling (edema)	Yes, skull fracture
Romner 2000[38]	Mild head injury: GCS 14–15, LOC for <20 min, absence of focal neurological deficits, and no signs of acute intracranial abnormality revealed by a CT scan.	EDH, SDH, SAH, brain contusion, brain edema	No
Thaler 2015[39]	MHI: a score of 13–15 on the GCS.	EDH, SDH, SAH, intracerebral bleeding	No
Welch 2016[40]	Blunt closed head injury, GCS 9–15.	Acute intracranial lesion is defined as any trauma induced or related finding and includes extra-axial lesions (acute EDH, SDH), cortical contusion, ventricular compression or trapping, brain herniation, intraventricular hemorrhage, hydrocephalus, SAH, petechial hemorrhagic or bland sheer injury, brain edema, post-traumatic ischemia, intracerebral hematoma, dural venous sinus injury, and/or thrombosis	No
Wolf 2013[41]	Blunt head trauma, GCS 13–15.	EDH, SDH, SAH, intracerebral hemorrhage, brain contusion	No
Zongo 2012[42]	Isolated head trauma, GCS 13–15 determined by the attending physician, and with one or more of the following risk factors: LOC, PTA, repeated vomiting, severe headache, dizziness, vertigo, alcohol intoxication, anticoagulation, and age >65 yrs	EDH, SDH, intracerebral hemorrhages, bland contusion, edema, pneumocephalus, skull fracture	Yes, skull fracture

ACEP/CDC, American College of Emergency Physicians/Centers for Disease Control and Prevention; EDH, epidural hematoma; EFNS, European Federation of Neurological Societies; GCS, Glasgow Coma Scale; ICH, intracerebral hemorrhage; LOC, loss of consciousness; MHI, mild head injury; MHT, mild head trauma; mTBI, mild traumatic brain injury; NR, not reported; PTA, post-traumatic amnesia; SAH, subarachnoid hemorrhage; SDH, subdural hematoma; SNC, Scandinavian Neurotrauma Committee; TBICDE Neuroimaging WG, Traumatic Brain Injury Common Data Elements Neuroimaging Working Group.
